# Identification and evaluation of phenotypic characters and genetic diversity analysis of 1,558 foxtail millet germplasm resources for conservation and breeding

**DOI:** 10.3389/fpls.2025.1624252

**Published:** 2025-07-16

**Authors:** Xinwei Xue, Zhikun Yu, Ankang Mu, Fan Yang, Dan Liu, Shi Zhang, Jialin Zhang, Yao Cheng, Yushan Zhao, Yongping Zhang, Xianrui Wang

**Affiliations:** ^1^ College of Agronomy,Inner Mongolia Agricultural University, Hohhot, Inner Mongolia, China; ^2^ Chifeng Academy of Agricultural and Animal Husbandry Sciences, Chifeng, Inner Mongolia, China

**Keywords:** foxtail millet, germplasm resources, agronomic traits, comprehensive evaluation, genetic diversity analysis

## Abstract

Foxtail millet (*Setaria italica*) is a specialty mixed grain crop that originated in China. This study comprehensively assessed the phenotypic variability of 1,558 foxtail millet accessions to explore their genetic diversity and facilitate effective germplasm conservation. A total of 25 traits, including 11 quantitative and 14 qualitative, were investigated based on the quantization of physical and chemical descriptors and digital image analysis. The findings revealed significant variations in the coefficient of variation (CV) among the quantitative traits, which ranged from 3.90–28.38%. That of the qualitative traits ranged from 7.44–66.20%. The Shannon-Wiener Index (H′) of the quantitative traits ranged between 1.86–2.08, and that of the qualitative traits ranged from 0.04–1.40. High genetic diversity was also detected among the 1,558 accessions. Based on hierarchical clustering, 1,558 accessions were separated into five categories. The principal component analysis (PCA) results indicated that 10 principal components were extracted when the cumulative contribution rate of the phenotypic traits reached 64.30%. The comprehensive evaluation F-values calculated based on correlation and PCA analyses of 25 phenotypic traits were applied to all the accessions, and the top 10 varieties were identified. Collectively, this study showed the rich genetic diversity of the 1,558 foxtail millet accessions, which could provide a baseline for breeding new millet varieties.

## 1 Introduction

Foxtail millet (*Setaria italica*), an indigenous pseudo-cereal crop originating in China, was domesticated approximately 11,000 years ago from wild green foxtail (*Setaria viridis*) in northern China. This crop has been instrumental in shaping Chinese agricultural civilization and advancing dryland farming systems (Diao, 2019; Lv, 2018). Foxtail millet is a dual-purpose crop with significant potential for dietary improvement, resource conservation, and environmental sustainability. Nutritionally, it provides a well-balanced composition of essential micronutrients and bioactive compounds. As forage, it exhibits favorable characteristics, including high palatability, superior digestibility, and elevated crude protein content in aerial biomass (He et al., 2023; Jia et al., 2013; Jia and Diao, 2017). Furthermore, its distinctive agronomic traits, including short growth duration, exceptional water-use efficiency, and superior drought resistance, make it a cornerstone crop for sustainable dryland agroecosystems (Diao, 2017; Pardo and VanBuren, 2021). China maintains a foxtail millet cultivation area of 1.3 million hectares annually, producing over 2 million metric tons of foxtail millet, with world-leading productivity. The Inner Mongolia–Hebei–Shanxi production belt, which accounts for 67.1% of the national output through concentrated cultivation in these three provinces, has emerged as a characteristic agroecological zone for premium millet production (Yang et al., 2022, 2024).

Germplasm resources constitute the fundamental basis for genetic improvement and cultivar innovation of foxtail millet plants. China’s National Genebank currently conserves 28,915 accessions, representing 70% of the global foxtail millet germplasm, with remarkable genetic diversity documented through recent genomic surveys (Jia and Diao, 2022; Li et al., 2021). Contemporary breeding practices constrained by climate variability, agricultural intensification, and market-driven selection pressures have led to excessive reliance on narrow founder germplasm. Genetic erosion has resulted in diminished allelic diversity in modern cultivars, posing a significant threat to the species’ genetic reservoir (Jin et al., 2025). The systematic characterization and innovative utilization of foxtail millet germplasm have become critical priorities. Recent advances in high-throughput phenotyping and genomic selection have enabled more efficient germplasm exploitation, as evidenced by recent studies (Chen et al., 2023; Costa et al., 2017; Zhang et al., 2025; Zhou et al., 2015). Phenotypic diversity analysis enables the comprehensive characterization of germplasm attributes and elucidation of inheritance patterns, providing essential insights for deciphering crop domestication processes and informing precision breeding strategies. Huang et al. (2024) characterized 603 Shanxi accessions using 14 phenotypic parameters, revealing significant variation (CV=15.8–42.3%) and high Shannon diversity indices (H’=1.02–2.15), demonstrating the potential of this germplasm for broadening the genetic base of elite cultivars. Meng et al. (2024) identified two distinct ideotypes through multivariate analysis of 12 forage-related traits in 181 accessions: tall-stature types (180–220 cm) demonstrating high biomass yield (18–22 t/ha), and high leaf-stem ratio genotypes (>0.45) suitable for premium forage production. Xiang et al. (2020) conducted an ecogeographic characterization of 435 accessions across six agroecological zones, revealing significant (P<0.01) environment-trait interactions and distinct phenotypic adaptation patterns. Existing diversity studies have been constrained by regional sampling biases (68% from North China), limited germplasm representation (n<500), and incomplete trait coverage (≤20 traits). Nationwide investigations that systematically evaluate diverse germplasm types (landraces, cultivars, and elite lines) using comprehensive phenotyping (>25 traits) remain scarce. This study implemented a multidimensional evaluation framework incorporating 11 quantitative and 14 qualitative descriptors for 1,558 foxtail millet accessions (847 landraces, 432 breeding lines, and 256 cultivars). Advanced analytical approaches, including multivariate statistics, principal component analysis (PCA), and hierarchical clustering, were employed to: (1) establish comprehensive phenotypic profiles, (2) identify novel genetic variations, and (3) facilitate the strategic utilization of elite germplasm. These findings provide critical insights for enhancing genetic diversity in breeding programs and for establishing a foundation for the development of next-generation cultivars.

## 2 Materials and methods

### 2.1 Plant materials

A total of 1,558 foxtail millet accessions were obtained (**Supplementary Table 1**), including 847 local varieties, 432 species strains, 256 selected varieties, 4 genetic materials, and 19 other materials. The local varieties, species strains, genetic materials, and other materials were provided by the Academy of Agricultural and Animal Husbandry Sciences, Chifeng, Inner Mongolia, China. The selected varieties were provided by the National Millet Crops Research and Development System.

### 2.2 Experimental design

The experiment was carried out from 2023 to 2024 in the experimental demonstration base of the Academy of Agricultural and Animal Husbandry Sciences (41°51′ N; 119°08′ E), Chifeng, Inner Mongolia, China. The region is located in the northern part of the Yanshan Hills, with a temperate continental arid and semi-arid climate, average annual evaporation of 1600–2000 mm, frost-free period of 135–150 d, average annual temperature of 6.4 °C, and average altitude of 540 m above sea level. The previous crop at the experimental site in both years was corn. The experimental site featured sandy loam, the average soil organic matter content over the previous two years was 11.8 g·kg^-1^, alkaline dissolved nitrogen was 51.3 mg·kg^-1^, effective phosphorus was 22.8 mg·kg^-1^, quick-acting potassium was 86 mg·kg^-1^, and pH was 7.78.

**Figure 1:**
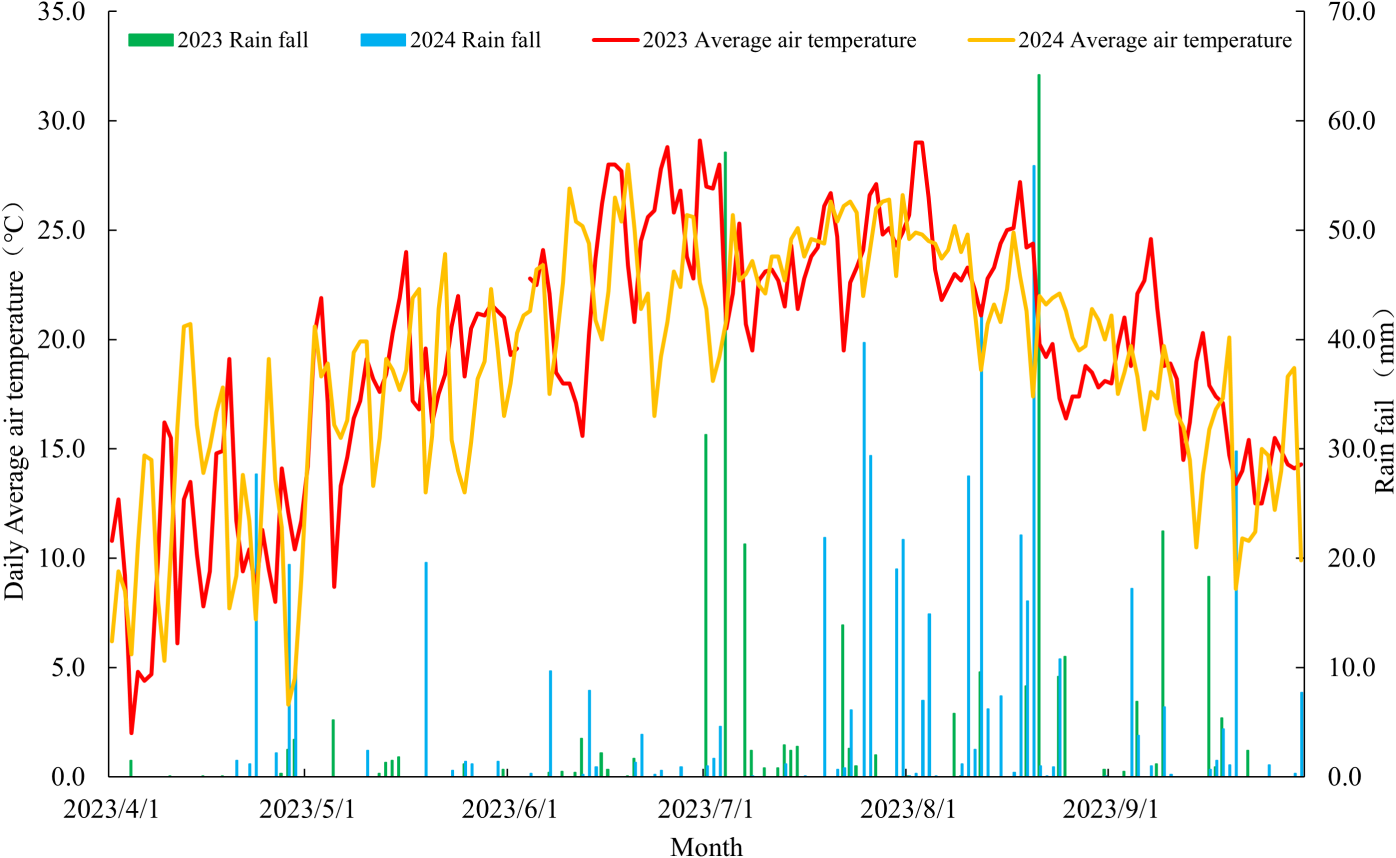
Temperature and precipitation during the growth and development of millet.

The trial employed a randomized complete block design with triplicate plots. Individual plots, which measured 10 m^2^ (2 m × 5 m), were established using Beidou satellite navigation-guided shallow-buried drip irrigation systems with alternating narrow-wide rows (60 + 40 cm). They featured 0.5 m average row spacing, 5 m row length, and four rows were cultivated per accessions. Mechanized furrow opening followed by manual seed broadcasting was performed on May 15, 2023, and May 23, 2024. A pre-sowing basal fertilization regime using NPK compound fertilizer (18–18–18) at 600 kg·ha^-^¹ was administered, with no supplementary fertilization throughout the cropping season. Seedling emergence was achieved through drip irrigation, followed by standardized thinning to maintain consistent plant density per meter. Scheduled irrigations (225 m³·ha^-^¹ per event) were implemented at five critical growth phases: seedling establishment, jointing, heading, anthesis, and grain filling. Harvesting operations were systematically performed on September 25, 2023, and October 1, 2024, corresponding to the physiological maturity stages of the plants. The agroclimatic parameters characterizing the 2023 and 2024 growing seasons are presented in **Figure 1**.

### 2.3 Selection and determination of phenotypic traits

A total of 25 representative phenotypic traits with relative genetic stability were investigated in 1,558 foxtail millet accessions, comprising 11 quantitative and 14 qualitative traits. Specific measurement methods for quantitative traits are provided in **Table 1**, and the evaluation criteria for qualitative traits are listed in **Table 2**. The qualitative traits were quantified and calculated according to the regulations (Lu, 2006; accessible via https://www.cgris.net). Both of which follow standardized quantification protocols. Observations were conducted on at least three individual plants per indicator and the average values were determined for quantitative traits. The investigation standards were established according to the “Descriptive Standards for Foxtail Millloet Germplasm Resources” (Lu, 2006; accessible via https://www.cgris.net) maintained by the National Crop Germplasm Resources Platform (National Crop Science Data Center). The final trait values were derived from the two-year average measurements of the obtained data.

**Table 1:** Quantitative traits and the associated test methods used in the experiment.

Quantitative traits	Measurement methods
Seedling-to-spike (STC) period	Days from emergence (DAE) to heading date
Growth period (GP)	DAE to physiological maturity (PM_90_)
Main spike length (MPL)	Length from the first spikelet insertion position to the panicle apex
Main stem length (MTL)	Length from the base of the first elongated internode on the main stem to the first spikelet insertion point at maturity
Stem thickness (ST)	Diameter at the midpoint of the basal elongated internode during maturity stage
Single-spike weight (SSW)	Air-dry weight of individual panicles at maturity stage
Single-spike grain weight (SGW)	Air-dried weight of grains from individual panicles
1000-grain weight (TGW)	Weight of 1000 mature seeds
Grain L*	Colorimetric analysis using 3NH NS800 spectrophotometer (D65 standard illuminant, 10° observer): L* value represents lightness of millet grain chroma
Grain a*	Colorimetric analysis using 3NH NS800 spectrophotometer (D65 standard illuminant, 10° observer): a* value represents Red-Green of millet grain chroma
Grain b*	Colorimetric analysis using 3NH NS800 spectrophotometer (D65 standard illuminant, 10° observer): b* value represents Yellow-Blue of millet grain chroma

**Table 2:** Qualitative traits and the associated test methods used in the experiment.

Qualitative trait	Measurement standard	Measurement method	Trait type
Leaf sheath color (LSC)	1.Green; 2.Red; 3.Violet	Colorimetric card	Multivariate traits
Seedling leaf color (SLC)	1.Green; 2. Yellow- Green	Colorimetric card	Multivariate traits
Stargazer color (SZ)	1.Yellow; 2.Green; 3.Violet	Colorimetric card	Multivariate traits
Spike shape (SS)	1.Fusiform; 2.Spikelet style; 3.Cat’s paw style; 4.Cylindrical style	Eye-measurement	Multivariate traits
Spike elasticity (SE)	1. Compact; 2. Intermediate; 3. Relaxed	Eye-measurement	Multivariate traits
Spike neck shape (SNC)	1. Erect; 2. Medium bend; 3. Hooked bend; 4. Curved	Eye-measurement	Multivariate traits
Seta lengths (SL)	1. Short; 2. Very short; 3. Long; 4. Very long	Eye-measurement	Multivariate traits
Bristle color (BC)	1.Yellow; 2.Green; 3.Violet	Colorimetric card	Multivariate traits
Anther color (AC)	1.White; 2.Orange; 3.Yellow	Colorimetric card	Multivariate traits
Hull color (HC)	1.White; 2.Orange; 3.Brown; 3.Black; 4.Red; 5.Yellow; 6.Cyan	Colorimetric card	Multivariate traits
Seedling leaf shape (SLS)	1. Semi-erect; 2. Erect; 3. Horizontal; 4. Downslope	Eye-measurement	Multivariate traits
Flowering leaf shape (FLS)	1. Erect; 2. Horizontal; 3. Downslope	Eye-measurement	Multivariate traits
Tillering character (TL)	1. Strong; 2. Moderate; 3. Weak	Eye-measurement	Multivariate traits
Lodging resistance character (LR)	1. Weak; 2. Very weak; 3. Moderate; 4. Strong; 5. Very strong	Eye-measurement	Multivariate traits

### 2.4 Calculation formula

The genetic diversity index (Shannon-Wiener index) was calculated by stratifying traits into 10 classes using the mean (X¯) and standard deviation (S). The classification ranged from class 1 (Xi ≤ X¯ - 2S) to class 10 (Xi ≥ X¯ + 2S), with 0.5 S intervals defining each class. Relative frequencies across classes were subsequently employed to derive the index values.


H'=−∑Pi×lnPi

Where Pi denotes the relative frequency corresponding to the i^th^ class of a given trait.

### 2.5 Statistical analysis

Data processing and analysis were performed using Microsoft Excel 2021, and principal component analysis was implemented using SPSS 26.0. GraphPad Prism was used to create correlation analysis plots and violin diagrams, followed by graphical integration using Adobe Illustrator 2025. Origin 2021 was used to conduct cluster analysis and generate corresponding visualizations.

## 3 Results

### 3.1 Genetic diversity analysis of quantitative traits

**Table 3** shows that the coefficient of variation (CV) for the 11 quantitative traits, which ranged from 3.90–28.38%. The grain L* exhibited the lowest CV (3.90), contrasting with the highest variability in single-spike grain, confirming substantial genetic diversity and stability within the 1,558 foxtail millet accessions. Shannon-Wiener index diversity indices (H’) spanned 1.86–2.08 across traits, with stem thickness displaying maximum genetic heterogeneity (H’=2.08), while grain b* showed constrained variation (H’=1.86), indicating trait-specific selection pressures.

**Table 3:** Analysis of quantitative traits of millet.

Traits	Mean	SD	Max.	Min.	Range	CV (%)	H’
Seedling-to-spike period	73.56	6.63	99.00	58.00	41.00	9.02	2.00
Growth period	111.99	4.63	126.00	102.00	24.00	4.14	2.02
Main spike length	29.06	5.32	51.63	12.60	39.03	18.29	2.05
Main stem length	129.51	20.04	184.13	67.50	116.63	15.47	2.07
Stem thickness	6.26	1.25	10.69	2.24	8.45	19.97	2.08
Single-spike weight	98.90	27.21	187.12	10.36	176.76	27.52	2.06
Single-spike grain weight	80.88	22.96	161.76	7.43	154.33	28.38	2.04
1000-grain weight	3.11	0.39	5.43	2.05	3.38	12.50	2.07
Grain L*	62.53	2.44	71.30	46.96	24.34	3.90	1.91
Grain a*	6.77	1.13	10.18	0.97	9.21	16.73	1.92
Grain b*	28.03	3.38	35.77	8.13	27.64	12.07	1.86

SD, Standard Deviation. Max, Maximum values. Min., minimum value. CV, coefficient of variation. H’, Shannon-Wiener Index.

### 3.2 Genetic diversity analysis of qualitative traits

**Table 4** demonstrates that the coefficient of variation (CV) for 14 qualitative traits in the foxtail millet accessions spanned 7.44–66.20%, revealing differential polymorphism across morphological characteristics. The spike neck shape exhibited the smallest CV (7.44%), whereas the spike shape exhibited the largest CV (66.20%). The Shannon-Wiener index (H’) for qualitative traits ranged from 0.04 to 1.40, with spike neck shape displaying the lowest diversity (H’ = 0.04) and lodging resistance traits recording the highest diversity (H’ = 1.40). These findings confirm the substantial inter-accession divergence and heterogeneous genetic architecture across the germplasm collection, indicating rich allelic diversity for trait improvement.

**Table 4:** Analysis of qualitative traits of millet.

Traits	CV (%)	H’	Distribution frequency						
			1	2	3	4	5	6	7
leaf sheath color	31.71	0.44	1035 (66.43%)	232 (14.89%)	291 (18.68%)				
Seedling leaf color	51.86	0.87	1306 (83.38%)	252 (16.17%)					
Stargazer color	55.66	0.66	989 (63.48%)	1 (0.06%)	568 (36.46%)				
Spike shape	66.20	0.89	1066 (68.42%)	235 (15.08%)	22 (1.41%)	235 (15.08%)			
Spike elasticity	41.25	1.04	513 (32.93%)	308 (19.77%)	737 (43.73%)				
Spike neck shape	7.44	0.04	1547 (99.29)	10 (0.64%)					
Seta color	54.62	0.79	1105 (70.92%)	135 (8.66%)	314 (20.15%)	4 (0.26%)			
Bristle color	24.16	0.79	94 (6.03%)	1051 (67.46%)	413 (26.51%)				
Anther color	42.91	1.08	569 (36.52%)	382 (24.52%)	607 (38.96%)				
Hull color	13.05	0.44	7 (0.45%)	29 (1.86%)	20 (1.28%)	9 (0.58%)	77 (4.94%)	1398 (89.73%)	18 (1.16%)
Seedling leaf shape	23.20	0.69	99 (6.35%)	1217 (78.11%)	227 (14.57%)	15 (0.96%)			
Flowering leaf shape	17.65	0.62	30 (1.93%)	352 (22.59%)	1176 (75.48%)				
Tiller	30.61	0.81	272 (17.46%)	191 (12.26%)	1095 (70.28%)				
Lodging resistance	37.11	1.4	199 (12.77%)	216 (13.86%)	146 (9.37%)	758 (48.65%)	239 (15.34%)		

CV, coefficient of variation. H’, Shannon-Wiener Index.

### 3.3 Multivariate analysis of phenotypic traits: principal components and correlation networks

#### 3.3.1 Principal component analysis

The cumulative contribution rate of the first 10 principal components reached 64.30%, with each exhibiting eigenvalues >0.95. It should be noted that individual trait metrics contain distinctive information, leading to substantial data loss when reduced to a limited number of principal components. The low inter-variable correlations also hinder effective capture by a limited set of principal components. Consequently, the analysis did not reach the conventional empirical threshold of 70%. Nevertheless, eigenvalues >0.95 maintain relevance in follow-up investigations. The top 10 factors were extracted, clustering traits with similar effects into distinct groups. This process transformed the 25 original traits into 10 novel independent composite indices. PC1 was predominantly governed by seedling-to-spike period, stem thickness, single spike weight, single spike grain weight, and stargazer color (contribution to variance: 13.25%). Being yield-associated traits, they warrant priority attention for high-yield foxtail millet cultivar development. PC2 was primarily driven by colorimetric parameters L*, a*, b*, and seedling leaf color (contribution: 9.08%). As essential quality evaluation indices, they represent pivotal targets for quality improvement breeding. PC3 was chiefly influenced by main spike length, spike morphology, spike elasticity, flowering leaf shape, and stress tolerance (contribution: 7.99%). Being associated with growth resilience, corresponding indicators merit emphasis in breeding stress-tolerant foxtail millet varieties. PC4 was mainly governed by 1000-grain weight, seta color, and tillering capacity (contribution: 7.17%). PC5 and 6 were predominantly influenced by growth duration (contribution: 5.66%) and anther color and seedling leaf color (contribution: 4.62%), respectively. PC7 was largely defined by spike neck morphology and seta color (contribution: 4.42%). PC8, 9, and 10 were primarily associated with main stem length (contribution: 4.27%), leaf sheath coloration (contribution: 4.03%), and hull color (contribution: 3.82%), respectively. Traits underlying PCs 4–10 exhibit limited relevance to core contemporary breeding targets. Nevertheless, PC5 (growth duration) critically determines cultivar adaptability to cooler zones, enabling geographical expansion of millet cultivation. This parameter constitutes an essential consideration in breeding programs.

Component analysis revealed that the first 10 components cumulatively accounted for 64.30% of the total variance (eigenvalue threshold >0.95; **Table 5**). Dimensionality reduction consolidated the 25 original traits into 10 orthogonal principal components through eigenvalue decomposition. The results indicated that PC1 was primarily determined by seedling-to-spike period, stem thickness, single-spike weight, single-spike grain weight, and stargazer color, contributing 13.25% of the variance. PC2 was mainly composed of L*,a*, b*, and seedling leaf color, accounting for 9.08% of the variance. PC3 was principally defined by main spike length, spike shape, spike elasticity, flowering leaf shape, and anti-forgiveness, with a 7.99% contribution to variance. PC4 was characterized by 1000-grain weight, bristle color, and tiller, accounting for 7.17% of the variance. PC5, 6, and 7 were dominated by the growth period (5.66%), anther color and seedling leaf color (4.62%) and spike-neck shape and seta color (4.42%), respectively. PC8, 9, and 10 were principally associated with main stem length (4.27%), leaf sheath color (4.03%), and hull color (3.82%), respectively.

**Table 5:** Principle component analysis of phenotypic traits of millet accessions.

Traits	Principal component										
	1	2	3	4	5	6	7	8	9	10	Total load
Seedling-to-spike period	0.54	0.10	-0.20	-0.44	0.39	-0.09	0.04	-0.09	0.02	0.10	0.38
Growth period	0.10	-0.24	-0.17	-0.41	0.42	0.25	0.07	0.24	-0.14	0.01	0.13
Main spike length	0.37	-0.08	0.52	0.19	0.41	0.14	0.17	0.02	-0.11	0.05	1.67
Main stem length	-0.07	-0.13	0.29	-0.03	0.26	0.06	-0.37	0.43	0.20	0.21	0.86
Stem thickness	0.62	-0.19	-0.24	-0.10	0.21	-0.14	0.19	-0.03	0.05	0.11	0.49
Single-spike weight	0.84	-0.25	0.07	0.12	-0.07	0.06	-0.14	-0.14	0.25	0.07	0.83
Single-spike grain weight	0.81	-0.25	0.08	0.15	-0.11	0.06	-0.15	-0.16	0.27	0.06	0.76
1000-grain weight	0.21	-0.23	0.39	0.49	-0.22	0.08	0.02	0.19	-0.34	-0.08	0.50
Grain L*	0.16	0.49	-0.24	0.25	0.18	0.16	0.12	0.25	0.14	-0.12	1.39
Grain a*	0.32	0.71	-0.23	0.27	-0.01	0.07	-0.09	0.02	-0.06	0.00	0.99
Grain b*	0.36	0.77	-0.26	0.32	0.07	0.11	-0.01	0.12	-0.02	-0.04	1.41
leaf sheath color	-0.34	-0.25	-0.15	0.29	0.22	0.26	0.07	-0.09	0.45	-0.08	0.38
Seedling leaf color	0.21	0.43	0.36	-0.26	-0.17	-0.15	-0.07	-0.05	0.12	0.12	0.53
Stargazer color	-0.41	-0.05	-0.22	0.39	0.13	-0.17	0.06	-0.15	0.11	0.16	-0.15
Spike shape	0.14	-0.13	-0.42	-0.15	-0.32	0.01	0.13	0.24	0.22	0.10	-0.18
Spike elasticity	-0.07	0.21	0.44	0.02	0.36	-0.14	0.26	0.04	-0.11	-0.16	0.85
Spike neck shape	0.08	0.02	0.04	0.02	-0.31	-0.03	0.65	0.22	0.23	0.06	0.96
Seta color	-0.05	0.06	0.36	-0.10	-0.03	-0.19	0.45	0.11	0.21	0.04	0.85
Bristle color	-0.31	-0.26	-0.23	0.46	0.36	-0.01	0.05	-0.04	0.18	-0.12	0.08
Anther color	-0.07	-0.06	0.08	-0.08	-0.12	0.74	0.08	0.23	-0.08	0.24	0.97
Hull color	0.18	-0.03	0.11	-0.30	-0.10	0.29	0.01	-0.13	0.15	-0.78	-0.61
Seedling leaf shape	-0.07	0.09	-0.11	-0.05	0.00	0.38	0.29	-0.62	-0.20	0.26	-0.04
Flowering leaf shape	0.24	0.04	0.43	0.26	0.17	-0.02	0.04	-0.18	0.07	0.03	1.08
Tiller	0.39	-0.40	-0.16	0.40	-0.24	-0.06	0.06	0.09	-0.25	-0.05	-0.21
Lodging resistance	0.32	-0.29	-0.43	-0.01	0.19	-0.18	0.16	0.12	-0.33	-0.14	-0.58
Eigenvalue	3.31	2.27	2.00	1.79	1.41	1.16	1.11	1.07	1.01	0.95	
Contribution rate (%)	13.25	9.08	7.99	7.17	5.66	4.62	4.42	4.27	4.03	3.82	
Total account (%)	13.25	22.33	30.32	37.49	43.14	47.77	52.19	56.46	60.48	64.30	

**Figure 2:**
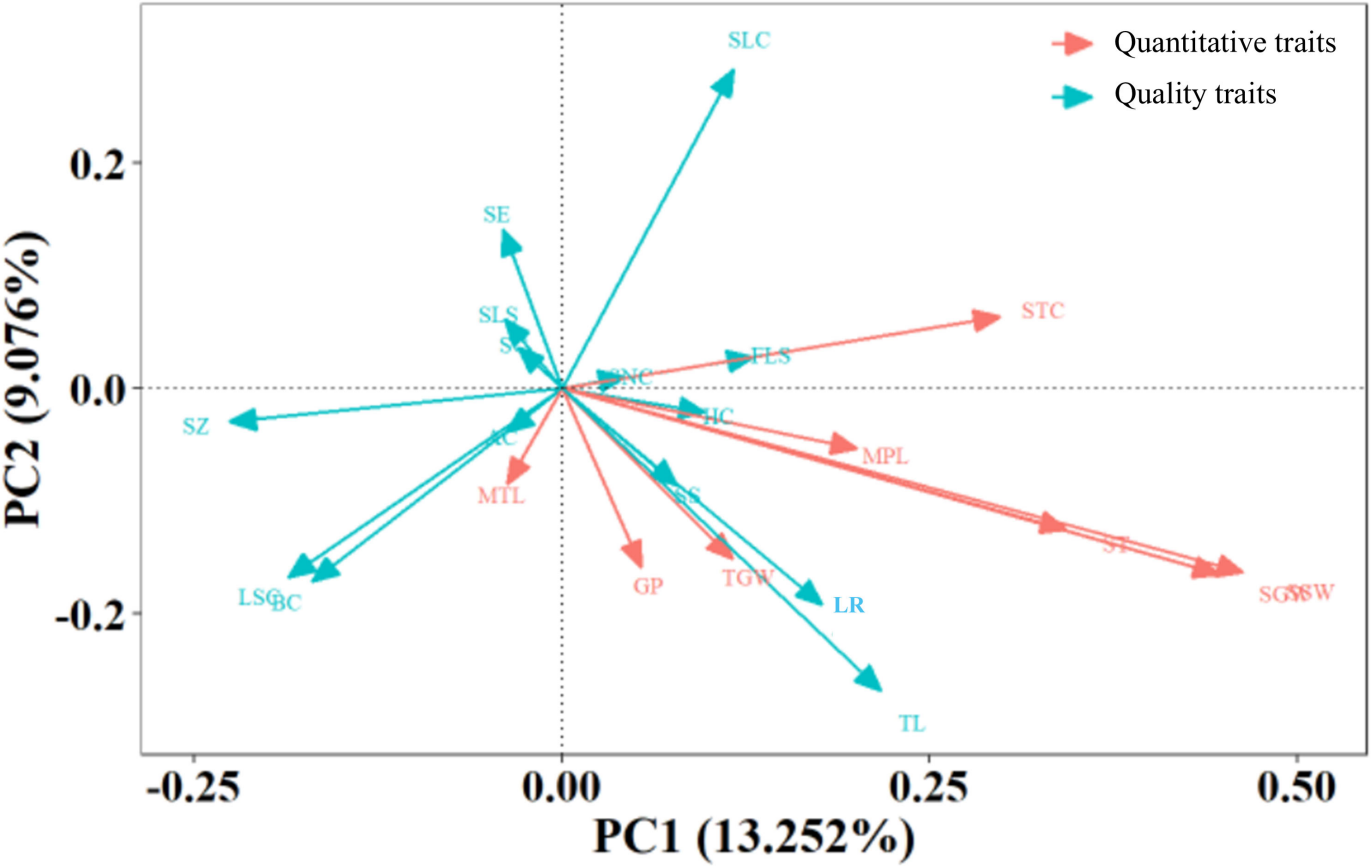
The principal component analysis of the 1,558 foxtail millet accessions.

The relationships between the trait indicators and principal components are shown (**Figure 2**). Among the quantitative traits, single-spike weight and single-spike grain weight contributed the most significantly to PC1 and served as crucial quantitative trait indicators. The growth period, 1000-grain weight, and main spike length showed similar directional trends as the single-spike weight and single-spike grain weight, constituting secondary quantitative trait indicators, indicating their positive correlation and synergistic effects on PC1. For qualitative traits, lodging resistance and tillers demonstrated positive correlations with PC1 and made the greatest contributions, serving as essential qualitative trait indicators. Spike shape and hull color exhibited directional proximity to lodging resistance and tillers, acting as secondary qualitative trait indicators.

#### 3.3.2 Correlation analysis

**Figure 3:**
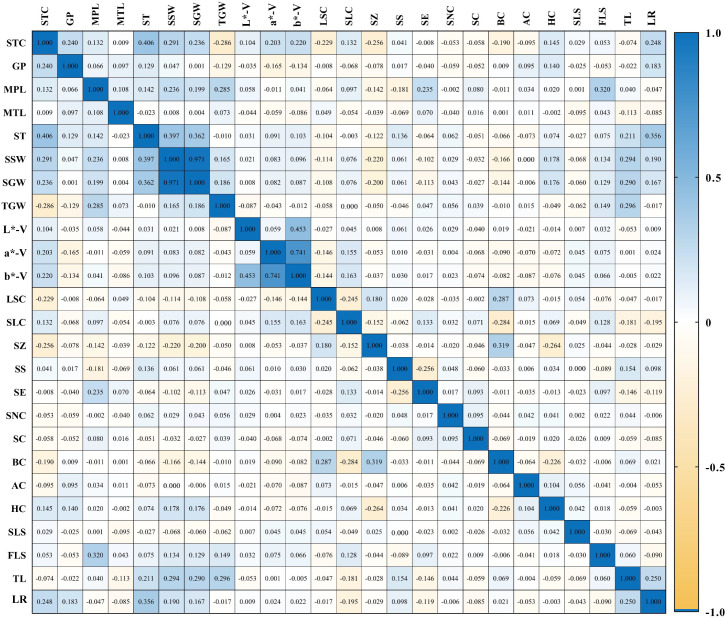
Correlation analysis of phenotypic traits in millet STC, seedling-to-spike period; GP, growth period; MPL, main spike length; MTL, main stem length; ST, stem thickness; SSW, single spike weight; SGW, single spike grain weight; TGW, 1000-grain weight; L-V, L*-V; a-V, a*-V; b-V, b*-V; LSC, leaf sheath color; SLC, seedling leaf color; SZ, stargazer color; SS, spike shape; SE, spike elasticity; SNC, spike neck shape; SC, bristle color; AC, anther color; HC, hull color; SLS, seedling leaf shape; FLS, flowering leaf shape; TL, tiller; LR, lodging resistance.

Correlation analysis was conducted on 11 quantitative and 14 qualitative traits across 1,558 foxtail millet accessions (**Figure 3**). The results demonstrated significant or highly significant correlations among most phenotypic traits; specifically, significant correlations were observed between single-spike weight and single-spike grain weight, as well as between grain a* and grain b. * Significant negative correlations were found between seedling-to-spike period and 1000-grain weight, and between seedling-to-spike period and leaf sheath color; weak correlations of varying degrees existed among other traits, indicating that most phenotypic traits in the foxtail millet accessions exhibited interactive and mutually reinforcing relationships.

A comprehensive evaluation system was established by first deriving the score coefficients for the 10 principal components using the eigenvector matrix and normalized data from 25 standardized phenotypic traits. Individual principal component scores were computed through dedicated formulae, exemplified by:

F1 = 0.298×STC+0.054×GP+0.201×MPL-0.037×MTL+0.343×ST+0.463×SSW+0.447×SGW+0.116×TGW+0.088×Grain L*+0.174×Grain a*+0.197× Grain b*-0.186×LSC+0.116×SLC-0.226×SZ+0.077×SS-0.040×SE+0.042×SNC-0.030×SL-0.169×BC-0.037×AC+0.097×HC-0.039×SLS+0.130×FLS+0.217×TL+0.177×LR.

Where STC is the Seedling-to-spike period, GP is the growth period, MPL is the main spike length, MTL is the main stem length, ST is the stem thickness, SSW is the single-spike weight, SGW is the single-spike grain weight, TGW is the 1000-grain weight, LSC is the leaf sheath color, SLC is the seedling leaf color, SZ is the stargazer color, SS is the spike shape, SE is the spike elasticity, SNC is the spike neck shape, SL is the seta length, BC is the bristle color, AC is the anther color, HC is the hull color, SLS is the seedling leaf shape, FLS is the flowering leaf shape, TL is the tillering character, and LR is the lodging resistance character.

The subsequent components were calculated using the coefficients listed in **Table 6**. The 12 principal component scores were normalized with factor-weighting coefficients calculated according to their contribution rates. This process yielded the final composite evaluation formula:

**Table 6:** Coefficients of 10 principal component scores.

Traits	Matrix of component score coefficients									
	1	2	3	4	5	6	7	8	9	10
Seedling-to-spike period	0.298	0.064	-0.144	-0.331	0.33	-0.086	0.042	-0.084	0.023	0.105
Growth period	0.054	-0.158	-0.12	-0.303	0.354	0.231	0.064	0.233	-0.142	0.008
Main spike length	0.201	-0.053	0.364	0.14	0.348	0.132	0.157	0.018	-0.107	0.054
Main stem length	-0.037	-0.085	0.206	-0.022	0.22	0.051	-0.349	0.412	0.2	0.217
Stem thickness	0.343	-0.125	-0.168	-0.071	0.179	-0.132	0.178	-0.024	0.049	0.11
Single-spike weight	0.463	-0.163	0.051	0.09	-0.057	0.055	-0.129	-0.134	0.249	0.07
Single-spike grain weight	0.447	-0.165	0.054	0.112	-0.095	0.055	-0.14	-0.15	0.267	0.058
1000-grain weight	0.116	-0.151	0.275	0.364	-0.185	0.071	0.022	0.181	-0.34	-0.082
Grain L*	0.088	0.324	-0.169	0.188	0.15	0.151	0.112	0.241	0.138	-0.125
Grain a*	0.174	0.469	-0.164	0.198	-0.006	0.062	-0.087	0.023	-0.056	-0.003
Grain b*	0.197	0.51	-0.182	0.242	0.055	0.103	-0.012	0.114	-0.022	-0.043
Leaf sheath color	-0.186	-0.167	-0.103	0.216	0.186	0.242	0.062	-0.088	0.448	-0.085
Seedling leaf color	0.116	0.283	0.254	-0.196	-0.146	-0.143	-0.064	-0.049	0.115	0.125
Stargazer color	-0.226	-0.03	-0.158	0.289	0.112	-0.162	0.056	-0.142	0.11	0.168
Spike shape	0.077	-0.086	-0.298	-0.113	-0.268	0.007	0.121	0.234	0.214	0.106
Spike elasticity	-0.04	0.142	0.312	0.014	0.301	-0.129	0.244	0.038	-0.105	-0.16
Spike neck shape	0.042	0.011	0.03	0.016	-0.261	-0.029	0.619	0.208	0.226	0.057
Seta color	-0.03	0.037	0.252	-0.075	-0.024	-0.173	0.424	0.109	0.209	0.045
Bristle color	-0.169	-0.172	-0.165	0.343	0.302	-0.012	0.047	-0.038	0.182	-0.12
Anther color	-0.037	-0.039	0.057	-0.056	-0.098	0.687	0.071	0.223	-0.076	0.247
Hull color	0.097	-0.021	0.079	-0.224	-0.085	0.265	0.01	-0.124	0.145	-0.795
Seedling leaf shape	-0.039	0.061	-0.078	-0.034	-0.002	0.356	0.273	-0.602	-0.202	0.261
Flowering leaf shape	0.13	0.028	0.306	0.195	0.14	-0.015	0.039	-0.173	0.067	0.032
Tiller	0.217	-0.268	-0.111	0.298	-0.2	-0.051	0.057	0.09	-0.25	-0.054
Lodging resistance	0.177	-0.192	-0.303	-0.005	0.158	-0.167	0.154	0.12	-0.329	-0.145


F=0.206×F1+0.141×F2+0.124×F3+0.111×F4+0.088×F5+0.072×F6+0.069×F7+0.066×F8+0.063×F9+0.059×F10

**Figure 4:**
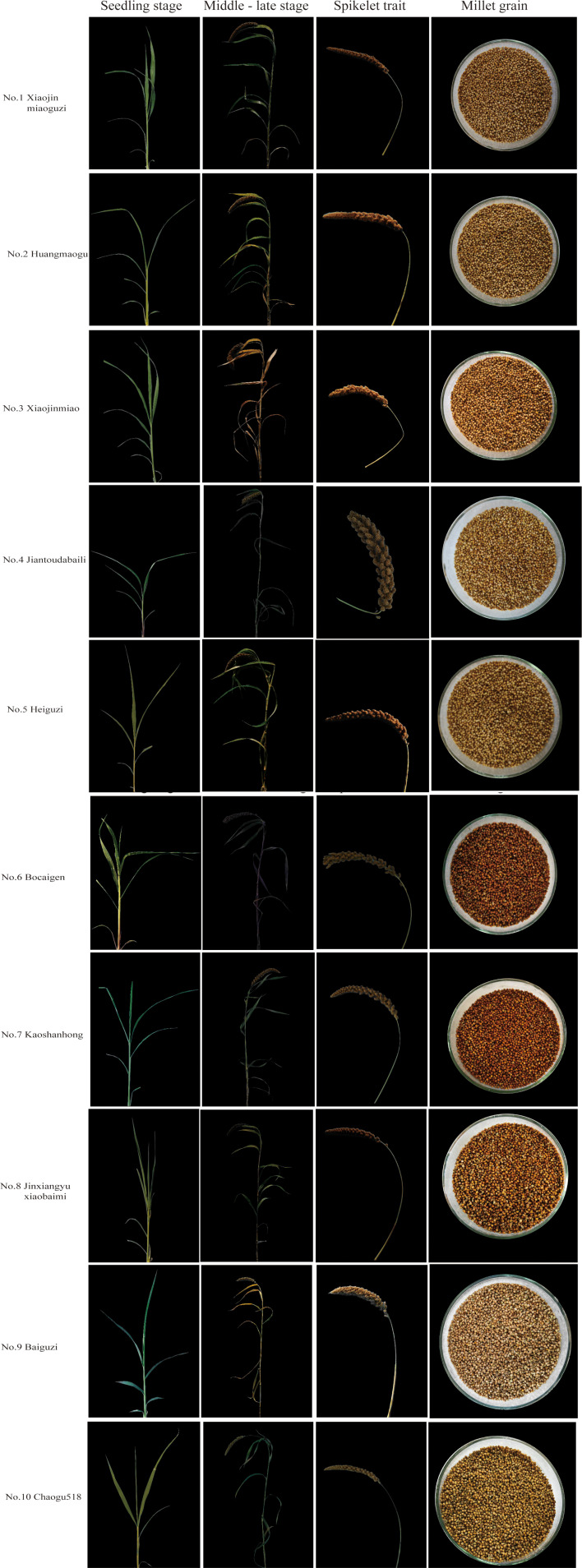
Millet accessions with top 10 comprehensive scores.

The 1,558 foxtail millet accessions were systematically ranked based on F-values, with the top 10 high-scoring accessions being selected (**Table 7**, **Figure 4**); detailed scores of other accessions are provided in the **Supplementary Table 1**. Elite accessions with superior composite scores exhibited significant advantages across qualitative traits, quantitative traits, and genetic attributes, establishing them as crucial foundational germplasm for future germplasm innovation and new cultivar breeding.

**Table 7:** Millet accessions with top 10 comprehensive scores.

Number	Name	Aggregate score	Seedling-to-spike period	Growth period	Main spike length	Main stem length	Stem thickness	Single-spike weight	Single-spike grain weight	1000-grain weight	Grain L*	Grain a*	Grain b*
148	Xiaojinmiaoguzi	0.933	71	106	30.5	134.13	8.17	102.88	88.8	3.14	64.19	7.5	31.41
116	Huangmaogu	0.850	72	110	32.63	92.66	6.61	119.04	100.47	3.19	63.2	7.53	29.86
136	Xiaojinmiao	0.814	69	107	32.27	92.94	7.52	124.3	102.82	3.33	63.53	6.57	28.97
978	Jiantoudabaili	0.798	77	115	32.27	136.6	7.84	132.16	101.35	2.94	65.27	7.1	31.57
98	Heiguzi	0.743	72	117	31.13	144.64	6.99	101.78	87.12	3.19	64.56	7.39	30.72
1507	Bocaigen	0.739	63	106	34.6	115.4	5.43	94.00	78.7	3.79	63.72	7.56	30.8
1152	Kaoshangong	0.715	70	114	39.03	151.24	6.32	107.19	89.29	3.53	66.61	4.46	24.3
224	Jinxiangyuxiaobaimi	0.703	72	110	24.77	113.14	6.56	90.63	76.56	3.16	65.26	9.21	32.33
995	Baiguzi	0.683	81	111	28.8	150.2	6.69	99.89	74.48	3.17	66.23	6.81	29.05
46	Chaogu518	0.654	72	106	32.27	116.14	7.92	117.74	100.39	3.5	59.05	6.92	26.39

### 3.4 Clustering pattern analysis in foxtail millet accessions

**Figure 5:**
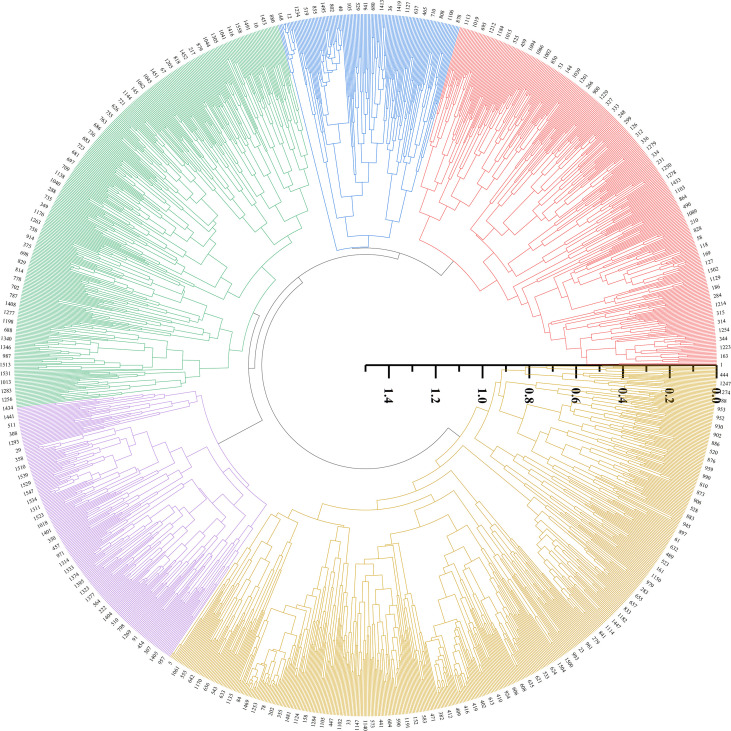
Cluster dendrogram of tested millet in accessions.

**Figure 6:**
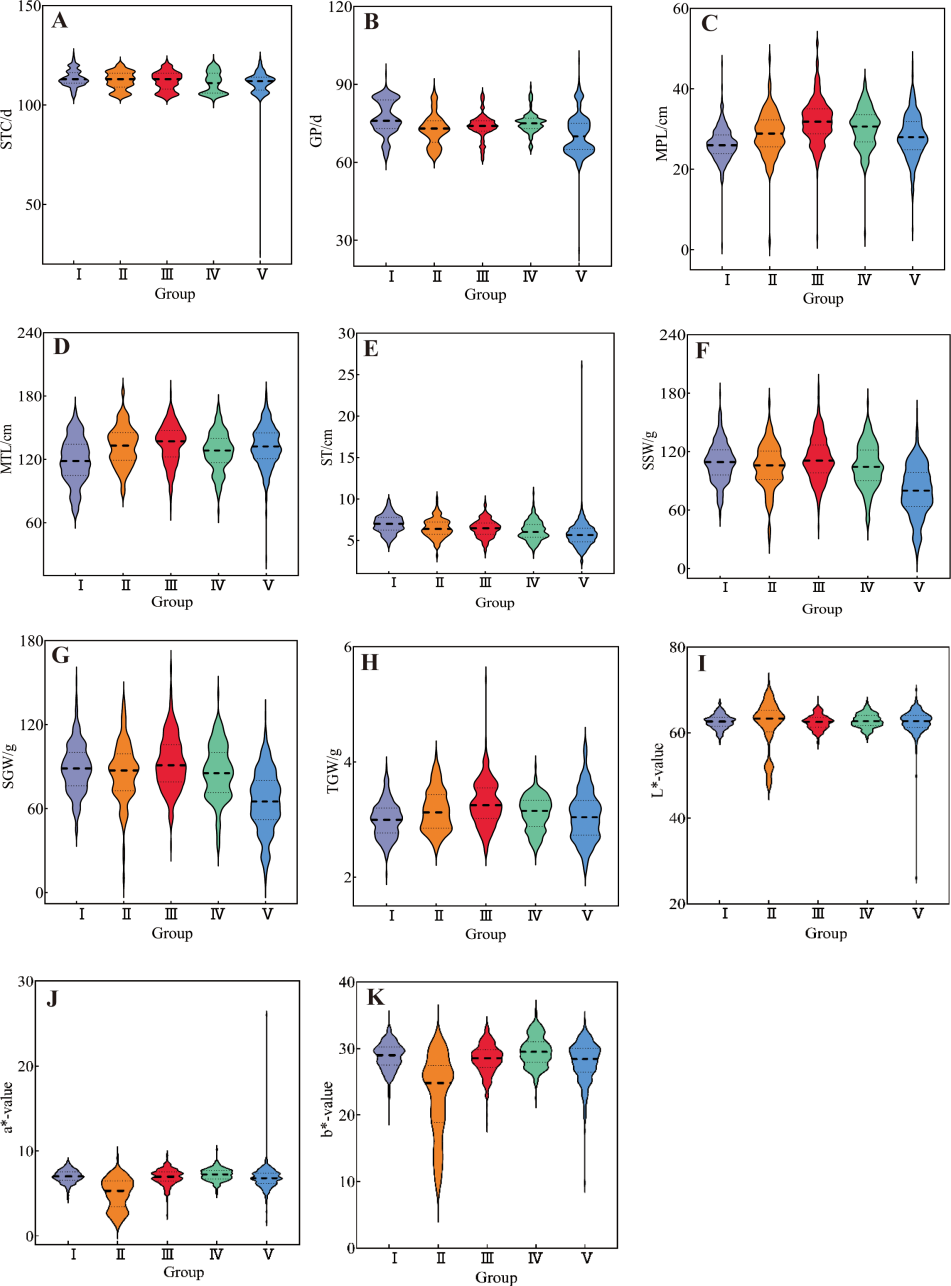
The violin diagram of 11 quantitative traits of millet accessions in five groups: **(A)** seedling-to-spike period, **(B)** growth period, **(C)** main spike length, **(D)** main stem length, **(E)** stem thickness, **(F)** single spike weight, **(G)** single spike grain weight, **(H)** 1000-grain weight, **(I)** L*-value, **(J)** a*-value, and **(K)** b*-value.

Cluster analysis was performed on 1588 foxtail millet accessions based on 24 phenotypic traits. The clustering divided them into five groups (**Figures 5**, **6**). Cluster I comprised 322 accessions, characterized by shorter main spike length (26.29 cm), shorter main stem length (118.55 cm), thicker stem diameter (6.99 mm), longer seedling-to-spike period (77.20 d), extended growth period (113.43 d), and lighter 1000-grain weight (2.99 g). These associated traits are all related to stress resistance in foxtail millet, providing abundant foundational materials for breeding stress-resistant varieties. Cluster II included 260 accessions, characterized by lower grain L* (61.72), a* (4.94), and b* (22.85). This group exhibits inferior grain quality, but can effectively broaden the genetic basis of quality-related accessions. Cluster III contained 283 accessions, characterized by longer main spike length (32.09 cm), heavier single-spike grain weight (92.56 g), heavier single-spike weight (112.9 g), and heavier 1000-grain weight (3.27 g), These associated traits all relate to yield components of foxtail millet, and can be prioritized as parental materials for high-yield breeding. Cluster IV included 213 accessions, characterized by shorter growth period (111.21 d), higher grain L* (62.83), a* (7.21), and b* (29.57). These indicators combine early maturity and high quality, providing abundant foundational materials for premium early-maturing varieties. Cluster V included 536 accessions, characterized by lighter single-spike grain weight (65.26 g), lighter single-spike weight (79.63 g), thinner stem diameter (5.66 mm), and shorter seedling-to-spike period (70.20 d).

## 4 Discussion

Systematic phenotyping remains the cornerstone methodology for germplasm characterization, providing essential biological insights that are unobtainable through molecular approaches alone. Empirical evidence has demonstrated that strategic phenomic data mining coupled with multi-trait evaluation enables the establishment of optimized core collections and improves breeding efficiency through enhanced genetic recombination potential.

### 4.1 Genetic diversity profiling in foxtail millet accessions

Phenotypic variability and diversity serve as crucial indicators of germplasm genetic diversity, directly manifesting as trait differentiation among accessions and revealing inheritance patterns. Enhanced variability and diversity correspond to increased interspecific divergence and genetic diversity (Hedari et al., 2019; Li et al., 2023a; Mirheidari et al., 2020). The coefficient of variation (CV) quantifying phenotypic dispersion revealed 11 quantitative traits in 1,558 foxtail millet accessions, ranging from 3.90% (grain L*) to 28.38% (single-spike grain weight). Qualitative traits exhibited CVs between 7.44% (spike neck shape) and 66.20% (spike shape), collectively demonstrating substantial phenotypic variability within the germplasm collection. Germplasm genetic diversity underpins crop breeding programs, where elevated diversity indices reflect greater allelic variation and enriched polymorphisms, as established in previous studies (Hou et al., 2024; Li et al., 2023b; Tang et al., 2025). Quantitative traits exhibited Shannon-Wiener index diversity indices ranging from 1.86 (grain b*) to 2.08 (stem thickness), indicating a differential genetic architecture. Qualitative traits showed indices from 0.04 (spike neck shape) to 1.40 (lodging resistance), revealing pronounced inter-accession variation. The broad-spectrum genetic variability across both trait categories suggests extensive allelic diversity within the germplasm panel. Collectively, the reduced variability and diversity indices observed in qualitative versus quantitative traits suggest stronger selection signatures in qualitative characteristics during domestication, corroborating established breeding theories (Guo et al., 2024). The phenotypic richness of 1,558 accessions - encompassing breeding lines, improved cultivars, and genetic stocks mirrored their heterogeneous geographical origins. Strategic exploitation of this diversity pool promises to enhance genetic recombination potential and facilitate targeted breeding programs.

### 4.2 Integrated analysis of phenotypic traits in foxtail millet accessions: correlation networks and clustering patterns

Inter-trait correlation studies enable the assessment of pleiotropic effects on genetic gain, thereby informing multi-trait selection strategies for breeding programs. In the present study, most phenotypic traits in foxtail millet accessions showed weak correlations to varying degrees, indicating interactive and promotive relationships between traits. A strong positive correlation between single-spike grain weight and single-spike weight underscores their co-regulation as a key yield determinant, necessitating simultaneous selection for yield improvement breeding. Coordinated variation in grain color parameters (grain a* vs. grain b*: r=0.65) reflects biochemical linkages in pigment biosynthesis, making them pivotal selection markers for grain quality enhancement. The negative association (r=-0.53) between the vegetative phase duration (seedling-to-spike period) and 1000-grain weight implies developmental trade-offs, where the extended vegetative phase compromises grain-filling capacity, which is a critical consideration for yield component balancing. Strategic integration of trait correlation networks enables the identification of complementary germplasm combinations, facilitating the development of transgressive segregants in hybrid breeding (Jiao et al., 2021; Zhang et al., 2022; Zhou et al., 2021). Phenotypic clustering serves as an effective proxy for genomic divergence assessment, with dendrogram topology revealing phylogenetic affinities (Dalmaijer et al., 2022; Li et al., 2020; Runjić et al., 2025). This methodology has been extensively validated for major crops, including soybeans (*Glycine max*; Xu et al., 2022), rice (*Oryza sativa*; Jin et al., 2025), and wheat (*Triticum aestivum*; Zhang H. F. et al., 2023). Cluster analysis of 25 phenotypic traits from 1,558 foxtail millet accessions divided them into five clusters. Cluster III exhibited superior performance in terms of main spike length, single-spike weight, single-spike grain weight, main stem length, and 1000-grain weight, all of which are traits associated with yield components, making it a preferential parental material for high-yield breeding. Cluster IV showed a shorter growth period and advantages in grain L*, a*, and b* values. These traits were strongly correlated with grain quality, qualifying it as the preferred parental material for quality breeding. Cluster I performed poorly across all traits, whereas Clusters II and V showed intermediate performances.

### 4.3 Multivariate characterization of phenotypic traits in foxtail millet accessions: principal component analysis and synthetic evaluation

Principal component analysis (PCA), a dimensionality reduction technique extensively applied in germplasm evaluation, transforms multiple phenotypic traits into orthogonal principal components that effectively capture variance structures (Li et al., 2024; Nardo et al., 2005; Jolliffe and Cadima, 2016). The 25 phenotypic traits were condensed into 10 principal components (PCs) accounting for 64.30% of the cumulative variance. PC1 (13.25% variance) was predominantly loaded with developmental traits (seedling-to-spike period) and yield components (single-spike weight/grain weight). PC2 (9.08%) primarily reflected grain color parameters (L*, a*, and b*) and seedling pigmentation. High-loading traits (>0.75), including single-spike weight and seedling leaf color, emerged as key drivers of phenotypic variation. The integration of PCA with fuzzy membership functions enhances the germplasm assessment precision through multi-criteria optimization (Jang, 2021; Wang et al., 2025; Xue et al., 2024; Zhang Y. Z. et al., 2023). In this study, phenotypic data were first transformed using membership functions and then combined with the PCA results to calculate comprehensive F-scores. Higher F-scores indicated superior overall performance. The top ten accessions identified were Xiaojinmiao Guzi, Huangmaogu, Xiaojinmiao, Jiantou Dalibai, Heiguzi, Bocaign, Kaoshanhong, Jinxiangyu Xiaobaimi, Baiguzi, and Chaogu 518, demonstrating the advantages of qualitative traits, quantitative traits, and genetic characteristics, making them crucial foundation accessions for subsequent breeding. This study assessed the top-ranked 10 accessions exclusively using phenotypic performance data. All trials were performed in a single testing environment, lacking verification of genetic stability via molecular markers or multi-environment trials. Future work must strengthen data reliability through molecular marker analysis and expanded multi-location trials, thereby enhancing the general applicability of findings.

## 5 Conclusions

This study systematically characterized and evaluated 1,558 foxtail millet accessions based on phenotypic traits, providing critical insights for germplasm conservation and cultivar development. The germplasm collection demonstrated substantial polymorphism across the quantitative and qualitative trait categories. Integrated multivariate approaches (correlation networks, PCA, and hierarchical clustering) effectively characterized the genetic architecture, validating the robustness of the methodological framework. Multivariate evaluation using PCA-weighted F-value computation identified ten elite accessions with balanced trait superiority. Accessions showing transgressive segregation potential are recommended as core parental lines for hybridization breeding.

Nevertheless, the environmental sensitivity of phenotypic traits may compromise characterization reliability. Implementing multi-approach strategies for germplasm assessment could significantly improve the scientific rigor and precision of evaluation outcomes. Future studies will prioritize molecular marker profiling and genome-wide association analyses of these 1,558 foxtail millet accessions to establish comprehensive genetic characterization.

## Data Availability

The datasets presented in this study can be found in online repositories. The names of the repository/repositories and accession number(s) can be found in the article/**Supplementary Material**.
