# Supplementary material for: Identification and evaluation of phenotypic characters and genetic diversity analysis of 1,558 foxtail millet germplasm resources for conservation and breeding
Source: Front Plant Sci. 2025 Jul 16;16:1624252. doi: 10.3389/fpls.2025.1624252 (PMC12307377; doi:10.3389/fpls.2025.1624252)
Supplement: Supplementary file 1 [file Table1.docx]

Supplementary Material

**Supplementary table 1 The information of all 1558 germplasm sources** **and clustering and F-worth score**

| **Code** | **Germplasm name** | **Classification** | **Cluster** | **F-values** | **Code** | **Germplasm name** | **Classification** | **Cluster** | **F-values** |
| --- | --- | --- | --- | --- | --- | --- | --- | --- | --- |
| 1 | qitoubai | Local varieties | 1 | -0.156 | 780 | daobaqimaogu | Local varieties | 3 | -0.281 |
| 2 | hongmiaohonggu | Local varieties | 2 | 0.277 | 781 | zimiaomaogu | Local varieties | 5 | 0.024 |
| 3 | maobadouzi | Local varieties | 5 | 0.190 | 782 | tiejijugu | Local varieties | 5 | 0.144 |
| 4 | balixiang | Local varieties | 5 | 0.218 | 783 | qitouqing | Local varieties | 1 | -0.373 |
| 5 | huangzhangu | Local varieties | 5 | 0.252 | 784 | gagabaigu | Local varieties | 5 | -0.167 |
| 6 | dahongmiao | Local varieties | 5 | 0.362 | 785 | dalihuanggu | Local varieties | 5 | 0.093 |
| 7 | daobaqi | Local varieties | 5 | 0.577 | 786 | xiaohonggu | Local varieties | 5 | 0.226 |
| 8 | qibaitou | Local varieties | 3 | -0.016 | 787 | xiaowanbaigu | Local varieties | 3 | 0.138 |
| 9 | zhangsuidahongmiao | Local varieties | 5 | 0.188 | 788 | quezhayangu | Local varieties | 3 | 0.296 |
| 10 | xiaobaigu | Local varieties | 3 | 0.113 | 789 | daobaqi | Local varieties | 4 | -0.230 |
| 11 | hongmiaoyapoche | Local varieties | 5 | -0.169 | 790 | sanbiansegu | Local varieties | 3 | 0.354 |
| 12 | guanxuanqitoubai | Local varieties | 2 | 0.638 | 791 | erbaigu | Local varieties | 3 | 0.428 |
| 13 | changhong | Local varieties | 5 | -0.023 | 792 | shuigu | Local varieties | 5 | 0.005 |
| 14 | zibogen | Local varieties | 4 | -0.119 | 793 | dashengtougu | Local varieties | 4 | 0.233 |
| 15 | xiaohongmiaogu | Local varieties | 2 | 0.130 | 794 | chibaigu | Local varieties | 4 | 0.441 |
| 16 | chazihong | Local varieties | 5 | 0.092 | 795 | sizhihongmaosuigu | Local varieties | 5 | 0.470 |
| 17 | qitouqing | Local varieties | 3 | 0.062 | 796 | qitoubaihuapigu | Local varieties | 4 | -0.033 |
| 18 | qianchuanzi | Local varieties | 2 | 0.305 | 797 | huangmiaoerbaigu | Local varieties | 4 | -0.183 |
| 19 | dalaohuang | Local varieties | 2 | -0.117 | 798 | shanxibaigu | Local varieties | 4 | 0.422 |
| 20 | dabaigu | Local varieties | 5 | 0.021 | 799 | laolaisong | Local varieties | 1 | 0.167 |
| 21 | chaotianchu | Local varieties | 3 | -0.145 | 800 | xiaozigenhong | Local varieties | 3 | 0.206 |
| 22 | zhuyeqing | Local varieties | 5 | -0.259 | 801 | damaoguzi | Local varieties | 3 | -0.028 |
| 23 | dabaigu | Local varieties | 5 | -0.117 | 802 | huangmaogu | Local varieties | 2 | -0.512 |
| 24 | shanxibai | Local varieties | 3 | 0.196 | 803 | shengtoujin | Local varieties | 5 | 0.555 |
| 25 | qianchuanzi | Local varieties | 3 | 0.121 | 804 | hongmaoguzi | Local varieties | 3 | 0.333 |
| 26 | hongmiaoganjianzi | Local varieties | 3 | 0.180 | 805 | xiaobaigu | Local varieties | 5 | 0.189 |
| 27 | oulihong | Local varieties | 1 | -0.287 | 806 | maobaigu | Local varieties | 4 | 0.138 |
| 28 | niutougou | Local varieties | 1 | -0.383 | 807 | daimaoqinggu | Local varieties | 3 | 0.021 |
| 29 | jinxiangyuxiaosheng | Local varieties | 4 | 0.155 | 808 | maomaobaigu | Local varieties | 2 | -0.189 |
| 30 | tie807 | germplasm strain | 3 | -0.071 | 809 | zhangboxi | Local varieties | 3 | 0.108 |
| 31 | tie8412 | germplasm strain | 3 | -0.249 | 810 | tongmaolvgu | Local varieties | 5 | -0.048 |
| 32 | tie8050 | germplasm strain | 3 | -0.025 | 811 | huangmaogu | Local varieties | 3 | 0.315 |
| 33 | qisifengmaomaogu | Local varieties | 5 | 0.232 | 812 | dabaigu | Local varieties | 3 | 0.462 |
| 34 | tie8373 | germplasm strain | 2 | 0.553 | 813 | maomaohuangguzi | Local varieties | 3 | 0.306 |
| 35 | tie8337 | germplasm strain | 1 | -0.394 | 814 | maomaohuang | Local varieties | 3 | 0.381 |
| 36 | tuzizui | Local varieties | 2 | -0.126 | 815 | baiguzi | Local varieties | 3 | 0.032 |
| 37 | tie8080 | germplasm strain | 5 | -0.109 | 816 | xiaobaigu | Local varieties | 5 | 0.346 |
| 38 | tie8131 | germplasm strain | 1 | -0.488 | 817 | xiaoyingzigenhuanggu | Local varieties | 2 | -0.639 |
| 39 | 90-2601 | germplasm strain | 1 | -0.490 | 818 | qinggenbai | Local varieties | 3 | 0.366 |
| 40 | jikangwen5 | Local varieties | 2 | -0.257 | 819 | xiaohuanggu | Local varieties | 3 | -0.004 |
| 41 | jikangwen6 | Local varieties | 1 | -0.092 | 820 | baigu | Local varieties | 1 | 0.031 |
| 42 | naihanTG118 | Local varieties | 2 | -0.753 | 821 | 15qugu | Local varieties | 5 | 0.022 |
| 43 | chazihong | Local varieties | 5 | 0.335 | 822 | daqinggu | Local varieties | 1 | -0.042 |
| 44 | gongai2hao | selected variety | 3 | -0.327 | 823 | jinmiaobai | Local varieties | 4 | -0.137 |
| 45 | huangbacha | Local varieties | 4 | 0.263 | 824 | xiehuahuang | Local varieties | 5 | -0.313 |
| 46 | chaogu518 | germplasm strain | 2 | 0.654 | 825 | bianhanbai | Local varieties | 3 | 0.162 |
| 47 | chaogu108 | germplasm strain | 3 | 0.126 | 826 | bianhanhuang | Local varieties | 3 | 0.264 |
| 48 | xiaohonggu | Local varieties | 5 | -0.349 | 827 | dagenmiaoqitouhuanggu | Local varieties | 4 | 0.024 |
| 49 | nenxuanshiliu | selected variety | 1 | -0.205 | 828 | xiaohuanggu | Local varieties | 1 | -0.096 |
| 50 | gonggu74hao | selected variety | 1 | -0.306 | 829 | daihuanggu | Local varieties | 3 | 0.073 |
| 51 | gonggu75hao | selected variety | 1 | -0.086 | 830 | huangmaoshan | Local varieties | 3 | 0.257 |
| 52 | gongai5hao | selected variety | 1 | -0.633 | 831 | xiaoshanxihuang | Local varieties | 5 | 0.366 |
| 53 | gonggu63hao | selected variety | 1 | -0.133 | 832 | shanxihuang | Local varieties | 2 | -0.880 |
| 54 | gonggu66hao | selected variety | 4 | 0.220 | 833 | datouhuang | Local varieties | 5 | 0.129 |
| 55 | liaogu1hao | selected variety | 2 | -0.769 | 834 | jinmiaohuang | Local varieties | 1 | 0.064 |
| 56 | zhushami | Local varieties | 2 | 0.303 | 835 | huangshenggu | Local varieties | 5 | 0.214 |
| 57 | fenggu12hao | selected variety | 5 | 0.094 | 836 | qitouhuang | Local varieties | 4 | 0.073 |
| 58 | neigu4hao | selected variety | 1 | -0.478 | 837 | xinghuanggu | Local varieties | 5 | 0.229 |
| 59 | menggu12hao | selected variety | 1 | -0.188 | 838 | erhuanggu | Local varieties | 3 | 0.264 |
| 60 | mengfenggu7hao | selected variety | 1 | -0.046 | 839 | kaoshanhuang | Local varieties | 5 | -0.041 |
| 61 | jigu24 | selected variety | 5 | -0.265 | 840 | jinmihuang | Local varieties | 3 | 0.163 |
| 62 | jingu16hao | selected variety | 1 | -0.065 | 841 | jiansuihuang | Local varieties | 5 | 0.417 |
| 63 | jingu41hao | selected variety | 2 | -0.397 | 842 | qitoubai | Local varieties | 3 | -0.139 |
| 64 | dasansui | Local varieties | 5 | 0.350 | 843 | shanxibai | Local varieties | 2 | -0.724 |
| 65 | honggenguzi | Local varieties | 4 | 0.009 | 844 | suanpibai | Local varieties | 3 | 0.092 |
| 66 | zhaoyanggu | Local varieties | 3 | -0.059 | 845 | zigenbai | Local varieties | 1 | -0.013 |
| 67 | ji9806-1 | germplasm strain | 3 | -0.283 | 846 | laohanbai | Local varieties | 1 | -0.035 |
| 68 | dungu1haoxuanxi | germplasm strain | 1 | -0.316 | 847 | liushabai | Local varieties | 5 | -0.008 |
| 69 | huangpihuangjinmiao | Local varieties | 1 | -0.176 | 848 | gaojiebai | Local varieties | 5 | 0.001 |
| 70 | hongmigu | Local varieties | 2 | -0.134 | 849 | zuantianbai | Local varieties | 3 | -0.020 |
| 71 | liaogu5hao | selected variety | 4 | 0.102 | 850 | yucibai | Local varieties | 1 | -0.208 |
| 72 | jiugu16 | selected variety | 4 | 0.384 | 851 | dugenbai | Local varieties | 5 | 0.090 |
| 73 | jiugu20 | selected variety | 4 | 0.193 | 852 | dingxianbaigu | Local varieties | 2 | -0.681 |
| 74 | chanbi20-15B | germplasm strain | 1 | -0.375 | 853 | liushaobai | Local varieties | 5 | 0.043 |
| 75 | hongguzi-1 | Local varieties | 5 | 0.110 | 854 | laotoubai | Local varieties | 2 | -0.867 |
| 76 | laolaibianguzi | Local varieties | 5 | 0.005 | 855 | xisuibai | Local varieties | 2 | -0.882 |
| 77 | hongguzi | Local varieties | 5 | 0.034 | 856 | xiaoqinggu | Local varieties | 2 | -0.940 |
| 78 | hongguzi | Local varieties | 5 | 0.121 | 857 | daqinggu | Local varieties | 5 | 0.153 |
| 79 | hongguzi | Local varieties | 5 | 0.051 | 858 | qinggubian | Local varieties | 5 | 0.274 |
| 80 | hongguzi | Local varieties | 5 | -0.062 | 859 | jiehuaqing | Local varieties | 5 | 0.120 |
| 81 | hongguzi | Local varieties | 5 | 0.109 | 860 | qitouqing | Local varieties | 1 | -0.087 |
| 82 | hongguzi | Local varieties | 5 | -0.217 | 861 | damaqinggu | Local varieties | 5 | 0.014 |
| 83 | zhongnong21 | selected variety | 1 | -0.153 | 862 | hongmaoshan | Local varieties | 5 | 0.125 |
| 84 | hongguzi | Local varieties | 5 | -0.146 | 863 | hongmiaodaobaqi | Local varieties | 5 | -0.104 |
| 85 | shanxihonggu | Local varieties | 5 | -0.057 | 864 | zaoxinhong | Local varieties | 1 | -0.034 |
| 86 | hongguzi | Local varieties | 5 | -0.119 | 865 | hongdalangchui | Local varieties | 5 | 0.056 |
| 87 | hongguzi | Local varieties | 5 | -0.036 | 866 | yishenhong | Local varieties | 2 | -0.237 |
| 88 | hongguzi | Local varieties | 5 | 0.024 | 867 | hongqitou | Local varieties | 2 | -0.004 |
| 89 | hongguzi | Local varieties | 5 | -0.130 | 868 | hongmiaoqianchuan | Local varieties | 2 | 0.095 |
| 90 | erbaigu | Local varieties | 2 | -0.555 | 869 | dugenqitouhong | Local varieties | 3 | 0.180 |
| 91 | weizhi | Local varieties | 4 | 0.104 | 870 | dugenhong | Local varieties | 3 | 0.101 |
| 92 | zhangboyan | Local varieties | 2 | -0.071 | 871 | nverpang | Local varieties | 3 | 0.193 |
| 93 | xiaohuanggu | Local varieties | 5 | 0.171 | 872 | huanongsihao | Local varieties | 1 | -0.179 |
| 94 | weizhi | Local varieties | 4 | 0.135 | 873 | yanggu | Local varieties | 5 | 0.264 |
| 95 | zhushaguzi | Local varieties | 4 | 0.321 | 874 | sanshizhun | Local varieties | 2 | -0.220 |
| 96 | baiguzi | Local varieties | 2 | 0.176 | 875 | laohuwei | Local varieties | 5 | -0.057 |
| 97 | lvguzi | Local varieties | 2 | -1.119 | 876 | sanbianchou | Local varieties | 5 | -0.034 |
| 98 | heiguzi | Local varieties | 2 | 0.743 | 877 | bayigu | Local varieties | 1 | -0.097 |
| 99 | huangjinmiao | Local varieties | 4 | 0.527 | 878 | xianiutuigu | Local varieties | 2 | -0.274 |
| 100 | maomaogu | Local varieties | 4 | 0.374 | 879 | yangbuken | Local varieties | 3 | 0.245 |
| 101 | jingu42 | selected variety | 3 | -0.212 | 880 | congxiaohei | Local varieties | 3 | 0.263 |
| 102 | chaogu58 | selected variety | 1 | -0.159 | 881 | laoxiangguan | Local varieties | 2 | -0.109 |
| 103 | yangu18 | selected variety | 3 | 0.152 | 882 | yapoche | Local varieties | 2 | -0.153 |
| 104 | 14H758 | germplasm strain | 1 | -0.225 | 883 | 811gu | Local varieties | 5 | 0.412 |
| 105 | weizhi | Other germplasm | 2 | 0.009 | 884 | daanchunwei | Local varieties | 5 | 0.242 |
| 106 | chi70—33 | germplasm strain | 1 | -0.042 | 885 | 60tianhaijia | Local varieties | 5 | -0.120 |
| 107 | chi71—26 | germplasm strain | 5 | 0.372 | 886 | momogu | Local varieties | 5 | -0.013 |
| 108 | maomaogu | Local varieties | 4 | 0.429 | 887 | shengergu | Local varieties | 5 | 0.240 |
| 109 | hongmaozhuazi | Local varieties | 1 | 0.121 | 888 | liutiaoqing | Local varieties | 5 | 0.236 |
| 110 | heiguzi | Local varieties | 4 | 0.079 | 889 | liulenggu | Local varieties | 5 | -0.042 |
| 111 | tiegu5hao | selected variety | 1 | -0.296 | 890 | mujiju | Local varieties | 5 | -0.186 |
| 112 | digu | selected variety | 1 | -0.297 | 891 | zigancao | Local varieties | 4 | 0.424 |
| 113 | tie8049 | germplasm strain | 4 | 0.307 | 892 | huanongsihao | Local varieties | 5 | -0.288 |
| 114 | tie8050 | germplasm strain | 3 | 0.059 | 893 | baigenmujiju | Local varieties | 5 | 0.168 |
| 115 | 90-2606 | germplasm strain | 1 | -0.043 | 894 | dayangweiba | Local varieties | 5 | 0.318 |
| 116 | huangmaogu | Local varieties | 2 | 0.850 | 895 | baigenliaojiangbai | Local varieties | 5 | -0.054 |
| 117 | jikangwen4 | Local varieties | 1 | -0.536 | 896 | xiaobaigu | Local varieties | 5 | 0.007 |
| 118 | shengu1hao | selected variety | 1 | -0.470 | 897 | honggenliushabai | Local varieties | 5 | 0.114 |
| 119 | gonggu65hao | selected variety | 1 | -0.327 | 898 | maohuanggu | Local varieties | 5 | -0.102 |
| 120 | gonggu68hao | selected variety | 1 | -0.199 | 899 | baimiaoshengu | Local varieties | 2 | 0.478 |
| 121 | gonggu71hao | selected variety | 1 | 0.010 | 900 | baimiaotugu | Local varieties | 1 | 0.022 |
| 122 | chaoxingu2hao | selected variety | 1 | -0.387 | 901 | xiaobaigu | Local varieties | 5 | 0.280 |
| 123 | jingu29 | selected variety | 3 | 0.064 | 902 | baimiaoxiaoshenggu | Local varieties | 5 | 0.265 |
| 124 | longgu33 | selected variety | 3 | -0.010 | 903 | dayanggu | Local varieties | 5 | 0.584 |
| 125 | gongai6hao | selected variety | 3 | -0.034 | 904 | daguoliang | Local varieties | 1 | 0.172 |
| 126 | mengheigu8hao | selected variety | 1 | -0.540 | 905 | yanggu | Local varieties | 5 | 0.228 |
| 127 | jigu18 | selected variety | 1 | -0.381 | 906 | taogouhuang | Local varieties | 5 | 0.075 |
| 128 | jigu22 | selected variety | 1 | -0.239 | 907 | tietoupeng | Local varieties | 5 | 0.182 |
| 129 | ji200108-2 | germplasm strain | 1 | -0.129 | 908 | honggenmaogu | Local varieties | 5 | 0.209 |
| 130 | gonggu87hao | selected variety | 1 | -0.261 | 909 | baimiaoxiaoshenggu | Local varieties | 5 | 0.299 |
| 131 | gonggu88hao | selected variety | 1 | -0.333 | 910 | baigengu | Local varieties | 5 | 0.417 |
| 132 | JK007 | germplasm strain | 2 | 0.265 | 911 | 228-36 | germplasm strain | 4 | 0.166 |
| 133 | dajinmiao | Local varieties | 4 | 0.004 | 912 | dabaigu | Local varieties | 5 | 0.416 |
| 134 | longgu39 | selected variety | 1 | -0.163 | 913 | dabaigu | Local varieties | 3 | 0.113 |
| 135 | 1751-6 | germplasm strain | 3 | -0.234 | 914 | huangmaoguzi | Local varieties | 3 | 0.308 |
| 136 | xiaojinmiao | Local varieties | 2 | 0.814 | 915 | damaogu | Local varieties | 5 | 0.079 |
| 137 | chi158-17 | germplasm strain | 1 | -0.128 | 916 | xiaoyingsui | Local varieties | 3 | -0.165 |
| 138 | chi5702 | germplasm strain | 4 | -0.065 | 917 | daihuangmiao | Local varieties | 4 | 0.473 |
| 139 | JK008 | germplasm strain | 1 | -0.356 | 918 | hongmaolaoshu | Local varieties | 5 | 0.566 |
| 140 | gonggu84hao | selected variety | 1 | -0.875 | 919 | nanyangcaozigu | Local varieties | 3 | 0.127 |
| 141 | jibaimi1hao | selected variety | 5 | 0.024 | 920 | liulengguzi① | Local varieties | 5 | 0.081 |
| 142 | jigu22 | selected variety | 1 | -0.369 | 921 | fengqiugu | Local varieties | 3 | -0.102 |
| 143 | bao928 | selected variety | 1 | -0.336 | 922 | jinhuanghuang | Local varieties | 5 | 0.001 |
| 144 | jiugu11 | selected variety | 1 | -0.241 | 923 | liujiangcao | Local varieties | 5 | 0.260 |
| 145 | zhonggu9 | selected variety | 3 | -0.073 | 924 | xiaohongmiaogu | Local varieties | 5 | 0.109 |
| 146 | 60tianhaicang | Local varieties | 5 | 0.477 | 925 | hongmiaoxiaobaigu | Local varieties | 5 | 0.042 |
| 147 | shanxihonggu | Local varieties | 5 | 0.066 | 926 | yanxuncao | Local varieties | 5 | 0.256 |
| 148 | xiaojinmiaoguzi | Local varieties | 2 | 0.933 | 927 | hongmiaomujiju | Local varieties | 5 | -0.061 |
| 149 | dungu1hao | selected variety | 1 | -0.176 | 928 | laohuanggu | Local varieties | 5 | 0.278 |
| 150 | nonggu15 | selected variety | 2 | -0.225 | 929 | hongmiaoyangwenggu | Local varieties | 5 | 0.269 |
| 151 | jingu2hao | selected variety | 2 | -0.483 | 930 | guangtoumiaogenhong | Local varieties | 5 | 0.204 |
| 152 | jinbaoyuxiaobaimi（xifang） | Local varieties | 5 | 0.559 | 931 | hongyangweiba | Local varieties | 5 | 0.238 |
| 153 | jinbaoyuxiaobaimi（cusongyuan） | Local varieties | 5 | 0.454 | 932 | 228-26 | germplasm strain | 1 | 0.068 |
| 154 | menggu6hao | selected variety | 1 | -0.295 | 933 | bocaigen | Local varieties | 5 | 0.278 |
| 155 | jinfenggu | selected variety | 4 | 0.079 | 934 | niaojitou | Local varieties | 5 | -0.077 |
| 156 | xiaoxiangmi | Local varieties | 3 | -0.208 | 935 | tietoupeng | Local varieties | 2 | 0.090 |
| 157 | zhaonong21 | selected variety | 3 | 0.032 | 936 | tiaobanyue | Local varieties | 5 | 0.330 |
| 158 | pin194 | germplasm strain | 5 | 0.154 | 937 | xiaowenggu | Local varieties | 3 | 0.165 |
| 159 | pin196 | germplasm strain | 5 | -0.137 | 938 | daluochui | Local varieties | 5 | 0.378 |
| 160 | pin197 | germplasm strain | 1 | -0.099 | 939 | dabaigu | Local varieties | 5 | 0.167 |
| 161 | pin198 | germplasm strain | 5 | -0.105 | 940 | zhangjiahegu | Local varieties | 5 | 0.441 |
| 162 | pin199 | germplasm strain | 4 | -0.155 | 941 | honggendatougu | Local varieties | 2 | -0.021 |
| 163 | pin201 | germplasm strain | 1 | -0.348 | 942 | hongmiaoniaojiju | Local varieties | 5 | 0.143 |
| 164 | pin204 | germplasm strain | 5 | -0.261 | 943 | yigenjianxuanbian | Local varieties | 5 | 0.429 |
| 165 | pin205 | germplasm strain | 1 | -0.373 | 944 | liugenhong | Local varieties | 5 | 0.177 |
| 166 | pin206 | germplasm strain | 1 | -0.465 | 945 | xipixiaobaigu | Local varieties | 5 | 0.005 |
| 167 | pin207 | germplasm strain | 1 | -0.162 | 946 | huangzhangu | Local varieties | 5 | 0.059 |
| 168 | pin209 | germplasm strain | 1 | -0.307 | 947 | manqiangtazhangu | Local varieties | 5 | 0.215 |
| 169 | pin211 | germplasm strain | 1 | -0.550 | 948 | yumaohuangguzi | Local varieties | 5 | 0.394 |
| 170 | pin212 | germplasm strain | 1 | 0.043 | 949 | xiaomaogu | Local varieties | 5 | 0.180 |
| 171 | pin213 | germplasm strain | 2 | -0.429 | 950 | pihuyi | Local varieties | 5 | 0.259 |
| 172 | pin214 | germplasm strain | 4 | 0.207 | 951 | dangnianchen | Local varieties | 5 | -0.222 |
| 173 | heishatanxiaobaimi | Local varieties | 5 | 0.362 | 952 | huangzhangu | Local varieties | 5 | -0.041 |
| 174 | chao438 | germplasm strain | 3 | 0.154 | 953 | tiepachigu | Local varieties | 5 | 0.336 |
| 175 | chao637 | germplasm strain | 2 | -0.297 | 954 | huangzhangu | Local varieties | 5 | 0.139 |
| 176 | yangu16 | selected variety | 2 | -0.883 | 955 | baiguzi | Local varieties | 5 | 0.683 |
| 177 | jinxiangyuguzi | Local varieties | 5 | 0.012 | 956 | baizhangu | Local varieties | 5 | 0.055 |
| 178 | zhushagu | Local varieties | 2 | -0.053 | 957 | banmaoyihuang | Local varieties | 4 | 0.070 |
| 179 | long10-03018 | germplasm strain | 1 | 0.023 | 958 | xigenbai | Local varieties | 5 | -0.220 |
| 180 | jinxiangyuxiaobaimi | Local varieties | 3 | 0.652 | 959 | zhangu | Local varieties | 5 | 0.240 |
| 181 | long10-03020 | germplasm strain | 3 | 0.475 | 960 | bocaigenmaogu | Local varieties | 5 | 0.303 |
| 182 | long10-03021 | germplasm strain | 1 | -0.195 | 961 | liuyuexianguzi | Local varieties | 5 | 0.241 |
| 183 | long06-252 | germplasm strain | 1 | -0.116 | 962 | heizhangu | Local varieties | 1 | 0.171 |
| 184 | daihuangmigu | Local varieties | 1 | -0.102 | 963 | hongcaopigu | Local varieties | 5 | 0.316 |
| 185 | chazigu | Local varieties | 2 | 0.043 | 964 | hongguzi | Local varieties | 5 | 0.411 |
| 186 | long05-94130 | germplasm strain | 1 | -0.555 | 965 | zhanguzi | Local varieties | 5 | 0.259 |
| 187 | hongmaozi | Local varieties | 2 | -0.040 | 966 | yejiling | Local varieties | 5 | 0.024 |
| 188 | ji200106 | germplasm strain | 5 | 0.081 | 967 | longzhaogu | Local varieties | 5 | 0.368 |
| 189 | ji200108-2 | germplasm strain | 1 | -0.186 | 968 | longzhaogu | Local varieties | 3 | 0.226 |
| 190 | ziganguzi | Local varieties | 5 | 0.080 | 969 | maotigu | Local varieties | 1 | -0.075 |
| 191 | long10-03035 | germplasm strain | 3 | 0.163 | 970 | maoyugu | Local varieties | 1 | -0.289 |
| 192 | long10-03036 | germplasm strain | 3 | 0.108 | 971 | lushuibai | Local varieties | 4 | 0.403 |
| 193 | long10-03037 | germplasm strain | 3 | 0.051 | 972 | qingmiaozhangu | Local varieties | 4 | 0.362 |
| 194 | qichigao | Local varieties | 1 | -0.441 | 973 | tutizhangu | Local varieties | 3 | 0.244 |
| 195 | ji200314 | germplasm strain | 3 | -0.213 | 974 | yintianchou | Local varieties | 4 | -0.036 |
| 196 | ji87-1 | Other germplasm | 2 | -0.178 | 975 | youmaoxiehuahuang | Local varieties | 3 | 0.248 |
| 197 | 455hui | Other germplasm | 2 | -0.446 | 976 | dafuguzi | Local varieties | 3 | 0.500 |
| 198 | maozhuazixiaobaimi | Local varieties | 5 | 0.034 | 977 | daqinggengu | Local varieties | 4 | 0.008 |
| 199 | lvguzi-2 | Local varieties | 5 | -0.072 | 978 | jiantoudalibai | Local varieties | 5 | 0.798 |
| 200 | ji200307 | germplasm strain | 1 | -0.426 | 979 | dabagu | Local varieties | 5 | 0.479 |
| 201 | ji200316 | germplasm strain | 1 | -0.606 | 980 | xiaomaogu | Local varieties | 3 | 0.299 |
| 202 | hongguzi | Local varieties | 5 | -0.133 | 981 | wushisan | Local varieties | 4 | 0.060 |
| 203 | ji200115 | germplasm strain | 3 | 0.111 | 982 | kangjiangu | Local varieties | 5 | 0.416 |
| 204 | ji200118 | germplasm strain | 3 | 0.062 | 983 | xiaobagu | Local varieties | 5 | 0.137 |
| 205 | ji200103-4 | germplasm strain | 1 | -0.312 | 984 | laiwuxiaogu | Local varieties | 4 | -0.039 |
| 206 | pinxuan14hao | selected variety | 3 | 0.210 | 985 | laiwuhuang | Local varieties | 4 | 0.008 |
| 207 | pinxuan15hao | selected variety | 3 | 0.387 | 986 | hongmaogu | Local varieties | 3 | 0.077 |
| 208 | shengchanduizajiaogu | Local varieties | 5 | 0.152 | 987 | shengzitougu | Local varieties | 3 | 0.265 |
| 209 | guzibadouzi | Local varieties | 4 | 0.211 | 988 | qisuimaogu | Local varieties | 5 | 0.300 |
| 210 | pinxuan19hao | selected variety | 1 | -0.201 | 989 | jinmiaohuanggu | Local varieties | 5 | 0.090 |
| 211 | pinxuan21hao | selected variety | 1 | -0.152 | 990 | dajinxian | Local varieties | 5 | 0.265 |
| 212 | pinxuan22hao | selected variety | 1 | -0.230 | 991 | liangxiejian | Local varieties | 4 | 0.356 |
| 213 | pinxuan23hao | selected variety | 3 | -0.062 | 992 | taiandiao | Local varieties | 5 | 0.304 |
| 214 | pinxuan26hao | selected variety | 1 | -0.113 | 993 | bocaigen | Local varieties | 5 | 0.463 |
| 215 | heng201191 | germplasm strain | 1 | -0.370 | 994 | sanshizhun | Local varieties | 4 | 0.183 |
| 216 | heng201194 | germplasm strain | 1 | -0.262 | 995 | lvdouqiguzi | Local varieties | 5 | 0.426 |
| 217 | heng201195 | germplasm strain | 3 | 0.112 | 996 | 90-2601 | germplasm strain | 1 | -0.157 |
| 218 | heng2011122 | germplasm strain | 3 | 0.249 | 997 | 90-2603 | germplasm strain | 1 | -0.232 |
| 219 | heng2011342 | germplasm strain | 1 | 0.042 | 998 | 90-2306 | germplasm strain | 1 | -0.080 |
| 220 | heng2011343 | germplasm strain | 1 | -0.031 | 999 | 90-2614 | germplasm strain | 1 | -0.217 |
| 221 | heng2015S-10 | germplasm strain | 2 | -0.102 | 1000 | 90-2957 | germplasm strain | 1 | -0.298 |
| 222 | chiyoujingu | selected variety | 4 | -0.023 | 1001 | 90-2958 | germplasm strain | 1 | -0.454 |
| 223 | ziguzi | Local varieties | 1 | -0.263 | 1002 | 90-427 | germplasm strain | 1 | -0.312 |
| 224 | jinxiangyuxiaobaimi | Local varieties | 2 | 0.703 | 1003 | 90-435 | germplasm strain | 1 | -0.281 |
| 225 | chiguK1 | selected variety | 2 | -0.343 | 1004 | 90-487 | germplasm strain | 1 | -0.245 |
| 226 | 60tianhaicang | Local varieties | 4 | 0.077 | 1005 | 90-12226 | germplasm strain | 5 | 0.073 |
| 227 | chigu16 | selected variety | 3 | -0.181 | 1006 | 90-1414 | germplasm strain | 5 | -0.250 |
| 228 | chi5422 | germplasm strain | 3 | -0.275 | 1007 | 90-1438 | germplasm strain | 5 | -0.235 |
| 229 | chi5231 | germplasm strain | 3 | -0.032 | 1008 | 90-1648 | germplasm strain | 3 | -0.204 |
| 230 | chi2114 | germplasm strain | 3 | -0.025 | 1009 | 89-023 | germplasm strain | 1 | -0.305 |
| 231 | chi2707 | germplasm strain | 1 | 0.105 | 1010 | 89-027 | germplasm strain | 3 | -0.277 |
| 232 | chi2218 | germplasm strain | 1 | -0.077 | 1011 | 89-028 | germplasm strain | 3 | -0.124 |
| 233 | chi205-24 | germplasm strain | 3 | 0.147 | 1012 | 89-034 | germplasm strain | 1 | -0.393 |
| 234 | chi53-35 | germplasm strain | 1 | -0.456 | 1013 | 89-093 | germplasm strain | 3 | -0.070 |
| 235 | maozhuazishenhuangke | Local varieties | 3 | 0.330 | 1014 | 89-363 | germplasm strain | 1 | 0.004 |
| 236 | chi212-30 | germplasm strain | 5 | -0.053 | 1015 | 89-1302 | germplasm strain | 1 | -0.215 |
| 237 | chigu24 | germplasm strain | 3 | -0.187 | 1016 | 89-1384 | germplasm strain | 5 | -0.082 |
| 238 | chigu25 | germplasm strain | 2 | -0.400 | 1017 | 89-1616 | germplasm strain | 5 | -0.379 |
| 239 | chigu26 | germplasm strain | 5 | 0.093 | 1018 | 89-1853 | germplasm strain | 4 | 0.293 |
| 240 | chigu27 | germplasm strain | 2 | -0.177 | 1019 | 89-1616 | germplasm strain | 1 | -0.446 |
| 241 | fenghong5hao | selected variety | 1 | -0.127 | 1020 | 90-1628 | germplasm strain | 5 | -0.024 |
| 242 | fenghong7hao | selected variety | 1 | -0.278 | 1021 | 91-20 | germplasm strain | 5 | -0.328 |
| 243 | jinxiangyuzazhu | Local varieties | 5 | 0.129 | 1022 | 91-878 | germplasm strain | 1 | -0.401 |
| 244 | fenghong9hao | selected variety | 1 | -0.258 | 1023 | 91-988 | germplasm strain | 3 | -0.061 |
| 245 | jinpingu3hao | selected variety | 1 | -0.289 | 1024 | 91-1054 | germplasm strain | 1 | -0.403 |
| 246 | jinpingu4hao | selected variety | 1 | -0.264 | 1025 | 91-1318 | germplasm strain | 1 | -0.298 |
| 247 | jinpingu5hao | selected variety | 3 | 0.102 | 1026 | 94-303 | germplasm strain | 1 | 0.037 |
| 248 | zhangnong39hao | selected variety | 1 | -0.327 | 1027 | 94-308 | germplasm strain | 1 | -0.374 |
| 249 | zhangnong47hao | selected variety | 2 | -0.418 | 1028 | 94-317 | germplasm strain | 1 | -0.023 |
| 250 | jingu56hao | selected variety | 1 | -0.202 | 1029 | 94-320 | germplasm strain | 3 | -0.330 |
| 251 | changsheng11 | selected variety | 1 | 0.002 | 1030 | 94-322 | germplasm strain | 1 | -0.270 |
| 252 | changsheng13 | selected variety | 3 | -0.058 | 1031 | 94-331 | germplasm strain | 1 | -0.144 |
| 253 | jigu14 | selected variety | 2 | -1.086 | 1032 | 94-344 | germplasm strain | 3 | -0.063 |
| 254 | jigu15 | selected variety | 5 | -0.196 | 1033 | 94-348-1 | germplasm strain | 3 | -0.041 |
| 255 | jigu16 | selected variety | 1 | -0.383 | 1034 | 94-349-7 | germplasm strain | 1 | 0.080 |
| 256 | jigu17 | selected variety | 1 | -0.329 | 1035 | 94-412 | germplasm strain | 4 | 0.194 |
| 257 | jigu18 | selected variety | 1 | -0.212 | 1036 | 94-429 | germplasm strain | 1 | -0.143 |
| 258 | jigu20 | selected variety | 1 | -0.417 | 1037 | 94-433 | germplasm strain | 5 | -0.085 |
| 259 | jigu21 | selected variety | 1 | -0.327 | 1038 | 9-442 | germplasm strain | 5 | -0.138 |
| 260 | ji10H174 | germplasm strain | 3 | -0.070 | 1039 | 94-449 | germplasm strain | 1 | -0.249 |
| 261 | ji1102-3-2-2-2-3-10 | germplasm strain | 3 | 0.013 | 1040 | 94-493 | germplasm strain | 3 | -0.113 |
| 262 | ji1103-1-1-3-3-3 | germplasm strain | 1 | -0.278 | 1041 | 94-495 | germplasm strain | 3 | -0.181 |
| 263 | ji1107-6-1-1-1-1-2 | germplasm strain | 1 | -0.351 | 1042 | 94-497 | germplasm strain | 3 | -0.283 |
| 264 | ji1157-2-1-1-1-1-3 | germplasm strain | 3 | -0.226 | 1043 | 94-501 | germplasm strain | 1 | -0.252 |
| 265 | ji1212-4-5-5-2-3 | germplasm strain | 1 | -0.273 | 1044 | 94-503 | germplasm strain | 3 | -0.143 |
| 266 | ji1214-1-1-2-8-4 | germplasm strain | 1 | -0.234 | 1045 | 94-504 | germplasm strain | 3 | 0.017 |
| 267 | ji1226-1-1-3-3-1 | germplasm strain | 1 | -0.280 | 1046 | 94-505 | germplasm strain | 3 | -0.152 |
| 268 | ji1230-4-2-2-3-1 | germplasm strain | 3 | -0.307 | 1047 | 94-507 | germplasm strain | 1 | -0.180 |
| 269 | jingu55hao | selected variety | 1 | -0.149 | 1048 | 95-21 | germplasm strain | 3 | -0.078 |
| 270 | jingu60hao | selected variety | 1 | -0.240 | 1049 | 95-22 | germplasm strain | 1 | -0.341 |
| 271 | jingu62hao | selected variety | 1 | -0.086 | 1050 | 95-29 | germplasm strain | 1 | -0.119 |
| 272 | taixuan22 | selected variety | 2 | -1.139 | 1051 | 95-77 | germplasm strain | 5 | 0.010 |
| 273 | taixuan26 | selected variety | 2 | -0.047 | 1052 | 95-78 | germplasm strain | 1 | -0.237 |
| 274 | jiugu23 | selected variety | 1 | 0.197 | 1053 | 95-91 | germplasm strain | 3 | -0.152 |
| 275 | jiugu25 | selected variety | 1 | -0.020 | 1054 | 95-100 | germplasm strain | 3 | -0.046 |
| 276 | jiugu27 | selected variety | 1 | -0.466 | 1055 | 95-121 | germplasm strain | 3 | 0.006 |
| 277 | nonggu1 | Other germplasm | 4 | 0.139 | 1056 | 228-20 | germplasm strain | 4 | 0.080 |
| 278 | longgu39 | selected variety | 1 | -0.311 | 1057 | 95-125 | germplasm strain | 1 | -0.240 |
| 279 | longgu41 | selected variety | 5 | 0.195 | 1058 | 95-131 | germplasm strain | 3 | 0.104 |
| 280 | jinfenggu | selected variety | 3 | -0.041 | 1059 | 95-201 | germplasm strain | 1 | -0.477 |
| 281 | 18-9016 | germplasm strain | 5 | 0.112 | 1060 | 95-204 | germplasm strain | 1 | -0.314 |
| 282 | 18-58516 | germplasm strain | 1 | 0.068 | 1061 | 95-206 | germplasm strain | 5 | -0.136 |
| 283 | 18-58530 | germplasm strain | 5 | 0.229 | 1062 | 95-207 | germplasm strain | 3 | -0.102 |
| 284 | 18-58542 | germplasm strain | 1 | 0.007 | 1063 | 95-211 | germplasm strain | 3 | -0.110 |
| 285 | longgu3 | selected variety | 4 | 0.064 | 1064 | 95-366 | germplasm strain | 5 | 0.032 |
| 286 | longgu8 | selected variety | 5 | -0.065 | 1065 | 91-1 | germplasm strain | 5 | -0.325 |
| 287 | longgu9 | selected variety | 3 | -0.232 | 1066 | 96-2 | germplasm strain | 1 | -0.271 |
| 288 | longgu10 | selected variety | 3 | -0.107 | 1067 | 96-4 | germplasm strain | 1 | -0.342 |
| 289 | longgu11 | selected variety | 3 | -0.290 | 1068 | 96-6 | germplasm strain | 1 | -0.136 |
| 290 | longgu13 | selected variety | 3 | -0.099 | 1069 | 96-7 | germplasm strain | 1 | -0.159 |
| 291 | longgu14 | selected variety | 3 | 0.033 | 1070 | 96-8 | germplasm strain | 1 | 0.065 |
| 292 | longgu15 | selected variety | 3 | -0.268 | 1071 | 96-10 | germplasm strain | 1 | -0.097 |
| 293 | jingu61 | selected variety | 1 | -0.413 | 1072 | 96-13 | germplasm strain | 5 | -0.088 |
| 294 | jinfen108 | selected variety | 1 | -0.421 | 1073 | 228-5 | germplasm strain | 4 | 0.244 |
| 295 | jinfen110 | selected variety | 1 | -0.228 | 1074 | 96-24 | germplasm strain | 5 | -0.395 |
| 296 | jigu35 | selected variety | 5 | 0.033 | 1075 | 96-25 | germplasm strain | 5 | -0.061 |
| 297 | jigu36 | selected variety | 1 | -0.111 | 1076 | 96-26 | germplasm strain | 3 | -0.142 |
| 298 | jigu40 | selected variety | 1 | -0.660 | 1077 | 96-29 | germplasm strain | 2 | -0.046 |
| 299 | jigu42 | selected variety | 1 | -0.213 | 1078 | 96-30 | germplasm strain | 3 | -0.152 |
| 300 | jigu43 | selected variety | 1 | -0.173 | 1079 | 96-32 | germplasm strain | 1 | -0.407 |
| 301 | jigu45 | selected variety | 5 | 0.001 | 1080 | 96-39 | germplasm strain | 1 | 0.119 |
| 302 | jigu46 | selected variety | 3 | -0.110 | 1081 | 96-42 | germplasm strain | 5 | -0.049 |
| 303 | 16K2555 | germplasm strain | 1 | -0.121 | 1082 | 96-68 | germplasm strain | 5 | -0.044 |
| 304 | 18K3307 | germplasm strain | 1 | -0.166 | 1083 | 96-100 | germplasm strain | 1 | -0.185 |
| 305 | 18H1665 | germplasm strain | 4 | 0.071 | 1084 | tao1 | Other germplasm | 5 | -0.054 |
| 306 | 2017K6539 | germplasm strain | 4 | 0.017 | 1085 | tao2 | Other germplasm | 3 | 0.103 |
| 307 | 2017K5943 | germplasm strain | 4 | -0.316 | 1086 | tao4 | Other germplasm | 3 | 0.000 |
| 308 | 2017K5853 | germplasm strain | 4 | 0.154 | 1087 | tao5 | Other germplasm | 5 | 0.101 |
| 309 | 2017K5786 | germplasm strain | 1 | -0.055 | 1088 | 88-1076 | germplasm strain | 1 | -0.274 |
| 310 | 2017K5791 | germplasm strain | 5 | 0.008 | 1089 | daqingmiao | Local varieties | 1 | -0.256 |
| 311 | 17H465 | germplasm strain | 1 | -0.379 | 1090 | qingmiaobocaigen | Local varieties | 5 | 0.299 |
| 312 | 17H469 | germplasm strain | 1 | -0.281 | 1091 | 87-666 | germplasm strain | 3 | -0.088 |
| 313 | 17H492 | germplasm strain | 1 | -0.434 | 1092 | 87-667 | germplasm strain | 4 | -0.162 |
| 314 | 17H507 | germplasm strain | 1 | -0.260 | 1093 | 87-723 | germplasm strain | 3 | -0.169 |
| 315 | 17H508 | germplasm strain | 1 | -0.510 | 1094 | 87-745 | germplasm strain | 1 | -0.124 |
| 316 | 2017K4751 | germplasm strain | 1 | -0.148 | 1095 | 87-777 | germplasm strain | 1 | -0.335 |
| 317 | 17H392 | germplasm strain | 1 | -0.378 | 1096 | 87-825 | germplasm strain | 1 | -0.153 |
| 318 | 2017K4107 | germplasm strain | 1 | -0.408 | 1097 | 87-840 | germplasm strain | 3 | -0.225 |
| 319 | 2017K6840 | germplasm strain | 1 | -0.096 | 1098 | 87-941 | germplasm strain | 1 | -0.339 |
| 320 | 17H313 | germplasm strain | 5 | -0.154 | 1099 | 85-1004 | germplasm strain | 1 | -0.137 |
| 321 | 2017K3388 | germplasm strain | 1 | -0.355 | 1100 | 85-1022 | germplasm strain | 1 | -0.276 |
| 322 | 2017K3408 | germplasm strain | 5 | 0.115 | 1101 | 84-218 | germplasm strain | 2 | -0.305 |
| 323 | 17H227 | germplasm strain | 3 | -0.219 | 1102 | 84-219 | germplasm strain | 5 | -0.370 |
| 324 | 17H323 | germplasm strain | 1 | -0.340 | 1103 | 84-442 | germplasm strain | 1 | -0.378 |
| 325 | 17H247 | germplasm strain | 1 | -0.011 | 1104 | 84-515 | germplasm strain | 3 | -0.127 |
| 326 | 17H368 | germplasm strain | 1 | -0.213 | 1105 | 84-601 | germplasm strain | 5 | 0.180 |
| 327 | 2017K3562 | germplasm strain | 1 | -0.169 | 1106 | 83-409 | germplasm strain | 2 | -0.172 |
| 328 | 17H264 | germplasm strain | 1 | 0.034 | 1107 | 81-001 | germplasm strain | 2 | -0.663 |
| 329 | 17H281 | germplasm strain | 1 | -0.260 | 1108 | 79-529 | germplasm strain | 5 | 0.033 |
| 330 | 2017K3669 | germplasm strain | 1 | -0.370 | 1109 | 76-4-5 | germplasm strain | 5 | -0.445 |
| 331 | 17H971 | germplasm strain | 3 | 0.233 | 1110 | 88zao1468 | germplasm strain | 5 | -0.115 |
| 332 | 17M1309 | germplasm strain | 1 | -0.424 | 1111 | 88zao1483 | germplasm strain | 3 | -0.285 |
| 333 | 17M2852 | germplasm strain | 1 | -0.353 | 1112 | 88zao1494 | germplasm strain | 1 | -0.278 |
| 334 | 17H910 | germplasm strain | 1 | -0.101 | 1113 | 87han757 | germplasm strain | 1 | -0.585 |
| 335 | 17H911 | germplasm strain | 1 | -0.310 | 1114 | 87han908 | germplasm strain | 5 | -0.151 |
| 336 | 17H929 | germplasm strain | 1 | -0.428 | 1115 | 86han513 | germplasm strain | 1 | -0.105 |
| 337 | 17H1139 | germplasm strain | 1 | -0.532 | 1116 | 85han514 | germplasm strain | 1 | -0.454 |
| 338 | 17H1141 | germplasm strain | 1 | -0.460 | 1117 | 85han995 | germplasm strain | 1 | -0.298 |
| 339 | 17H1104 | germplasm strain | 1 | -0.268 | 1118 | 85han1034 | germplasm strain | 1 | -0.205 |
| 340 | 17H1148 | germplasm strain | 1 | -0.612 | 1119 | 85han1079 | germplasm strain | 1 | -0.220 |
| 341 | 17H1150 | germplasm strain | 1 | -0.332 | 1120 | 84han217 | germplasm strain | 5 | -0.095 |
| 342 | canggu5hao | selected variety | 1 | -0.361 | 1121 | 84han398 | germplasm strain | 1 | -0.155 |
| 343 | canggu6hao | selected variety | 1 | -0.287 | 1122 | 84han411 | germplasm strain | 3 | -0.271 |
| 344 | canggu7hao | selected variety | 1 | -0.457 | 1123 | 84han526 | germplasm strain | 1 | -0.388 |
| 345 | canggu9hao | selected variety | 1 | -0.323 | 1124 | xiaobacha | Local varieties | 5 | 0.047 |
| 346 | yugu17 | selected variety | 1 | -0.273 | 1125 | 84han605 | germplasm strain | 5 | -0.030 |
| 347 | datong34 | selected variety | 1 | 0.010 | 1126 | 84han639 | germplasm strain | 5 | 0.029 |
| 348 | datong37 | selected variety | 3 | 0.011 | 1127 | 84han644 | germplasm strain | 2 | 0.293 |
| 349 | datong41 | selected variety | 3 | -0.023 | 1128 | 84han1102 | germplasm strain | 3 | -0.185 |
| 350 | 228-10 | germplasm strain | 4 | 0.054 | 1129 | 83han508 | germplasm strain | 1 | -0.165 |
| 351 | shanxihonggu | selected variety | 5 | -0.145 | 1130 | 83han513 | germplasm strain | 5 | 0.006 |
| 352 | 228-7 | germplasm strain | 3 | 0.318 | 1131 | 83han574 | germplasm strain | 3 | 0.051 |
| 353 | aogu8hao | selected variety | 4 | 0.091 | 1132 | 16-8 | germplasm strain | 2 | -0.947 |
| 354 | 228-2 | germplasm strain | 4 | 0.260 | 1133 | liushitianhaicang | Local varieties | 5 | 0.290 |
| 355 | aohonggu | selected variety | 5 | -0.279 | 1134 | 85r1164 | germplasm strain | 1 | -0.172 |
| 356 | fenggu11hao | selected variety | 3 | -0.328 | 1135 | 86r1531 | germplasm strain | 5 | 0.048 |
| 357 | mengxianghonggu | selected variety | 1 | -0.110 | 1136 | 86r1566 | germplasm strain | 1 | -0.388 |
| 358 | 228-1 | germplasm strain | 4 | 0.214 | 1137 | 89-36 | germplasm strain | 5 | -0.096 |
| 359 | yugu34hao | selected variety | 5 | 0.077 | 1138 | maozhaobaimi | Local varieties | 3 | -0.318 |
| 360 | yugu33hao | selected variety | 1 | -0.189 | 1139 | qingmiaozhangu | Local varieties | 5 | 0.218 |
| 361 | yugu32hao | selected variety | 1 | -0.066 | 1140 | hongmaozhuazi | Local varieties | 5 | -0.138 |
| 362 | yugu18hao | selected variety | 1 | -0.453 | 1141 | shanxibai | Local varieties | 3 | 0.321 |
| 363 | yugu36hao | selected variety | 1 | -0.301 | 1142 | dahongmiao | Local varieties | 5 | 0.621 |
| 364 | yugu35hao | selected variety | 4 | 0.124 | 1143 | chigu3hao | selected variety | 5 | -0.036 |
| 365 | chenggu13hao | selected variety | 1 | -0.167 | 1144 | chigu4hao | selected variety | 3 | -0.128 |
| 366 | 228-39 | germplasm strain | 4 | 0.220 | 1145 | yanpibao | Local varieties | 4 | -0.208 |
| 367 | chigu19 | selected variety | 1 | -0.183 | 1146 | xiaolihuang | Local varieties | 3 | -0.175 |
| 368 | fenghonggu4hao | selected variety | 1 | -0.098 | 1147 | dabacha | Local varieties | 5 | 0.260 |
| 369 | tiansu1hao | selected variety | 1 | -0.294 | 1148 | zuo19bacha | Local varieties | 5 | -0.008 |
| 370 | tiansu3hao | selected variety | 1 | -0.354 | 1149 | kaihuahuang | Local varieties | 3 | 0.160 |
| 371 | tiansu7hao | selected variety | 1 | -0.520 | 1150 | ganjianzi | Local varieties | 5 | 0.211 |
| 372 | 228-34 | germplasm strain | 4 | 0.071 | 1151 | jinxiangyu | Local varieties | 2 | 0.020 |
| 373 | baogu23 | selected variety | 1 | -0.398 | 1152 | kaoshanhong | Local varieties | 5 | 0.715 |
| 374 | tonggu1hao | selected variety | 1 | -0.129 | 1153 | dahongmiao | Local varieties | 5 | 0.496 |
| 375 | 228-43 | germplasm strain | 3 | -0.106 | 1154 | qingmiaozhangu | Local varieties | 5 | 0.171 |
| 376 | yugu6 | selected variety | 3 | -0.139 | 1155 | qingmiaozhangu | Local varieties | 5 | 0.332 |
| 377 | xingu6hao | selected variety | 4 | 0.232 | 1156 | dasui | Local varieties | 5 | 0.104 |
| 378 | 228-40 | germplasm strain | 4 | 0.377 | 1157 | hongmiaogangshengtou | Local varieties | 5 | 0.314 |
| 379 | fengyougu6hao | selected variety | 2 | -0.189 | 1158 | hegegu | Local varieties | 5 | 0.283 |
| 380 | jiugu33 | selected variety | 1 | -0.294 | 1159 | bangzihun | Local varieties | 5 | -0.084 |
| 381 | aihuidangdizhong | Local varieties | 5 | -0.182 | 1160 | qitouxiaobaimi | Local varieties | 3 | 0.013 |
| 382 | zhangu | Local varieties | 5 | -0.047 | 1161 | rulaisheng | Local varieties | 3 | -0.170 |
| 383 | laolaibi | Local varieties | 5 | 0.022 | 1162 | yapoche | Local varieties | 1 | 0.260 |
| 384 | baodiheerhao | Local varieties | 5 | 0.277 | 1163 | xiaoyuci | Local varieties | 2 | 0.194 |
| 385 | dangdixiaosuiguzi | Local varieties | 5 | -0.107 | 1164 | esiniu | Local varieties | 5 | 0.324 |
| 386 | xunkedaqingmiao | Local varieties | 5 | -0.269 | 1165 | yapoche | Local varieties | 2 | -0.009 |
| 387 | laolaibian | Local varieties | 5 | 0.218 | 1166 | hongmiaoxiaobaimi | Local varieties | 5 | 0.401 |
| 388 | shuangshou | Local varieties | 5 | -0.112 | 1167 | qitouxiaobaimi | Local varieties | 2 | -0.073 |
| 389 | aihuixiaolihuang | Local varieties | 5 | -0.062 | 1168 | tiebaliulengzi | Local varieties | 5 | 0.239 |
| 390 | zaopi | Local varieties | 5 | -0.549 | 1169 | baodigao | Local varieties | 4 | 0.042 |
| 391 | fudingzhu | Local varieties | 5 | -0.114 | 1170 | shanxibei | Local varieties | 5 | 0.168 |
| 392 | huangshagu | Local varieties | 5 | -0.141 | 1171 | rulaisheng | Local varieties | 3 | -0.096 |
| 393 | fudingzhu | Local varieties | 5 | -0.055 | 1172 | shengzijinxiaobaimi | Local varieties | 3 | 0.236 |
| 394 | aigu | Local varieties | 5 | 0.010 | 1173 | zhanguhun | Local varieties | 5 | -0.015 |
| 395 | shuilizhan | Local varieties | 5 | -0.064 | 1174 | daobaqi | Local varieties | 3 | -0.024 |
| 396 | gougenhong | Local varieties | 5 | -0.155 | 1175 | daqingmiaobaimi | Local varieties | 2 | 0.184 |
| 397 | zaopi | Local varieties | 5 | -0.382 | 1176 | qingmiaozuanzitou | Local varieties | 3 | -0.023 |
| 398 | huangshagu | Local varieties | 1 | -0.499 | 1177 | dabaigu | Local varieties | 3 | 0.092 |
| 399 | daobaqi | Local varieties | 5 | -0.337 | 1178 | yapoche | Local varieties | 1 | -0.032 |
| 400 | dangdizhong | Local varieties | 5 | -0.123 | 1179 | hongmiaodaobaqi | Local varieties | 5 | 0.304 |
| 401 | huangshagu | Local varieties | 5 | -0.304 | 1180 | qingmiaodaobaqi | Local varieties | 5 | 0.246 |
| 402 | huanggu | Local varieties | 5 | 0.109 | 1181 | hongmiaobaimi | Local varieties | 5 | 0.209 |
| 403 | dazaopi | Local varieties | 5 | -0.009 | 1182 | hongzhangu | Local varieties | 5 | 0.341 |
| 404 | huangshagu | Local varieties | 5 | 0.123 | 1183 | chazihong | Local varieties | 1 | 0.054 |
| 405 | gougen | Local varieties | 5 | -0.296 | 1184 | qianchuanzi | Local varieties | 1 | -0.353 |
| 406 | maodali | Local varieties | 5 | -0.185 | 1185 | xiaoqingmiao | Local varieties | 3 | -0.234 |
| 407 | 228-28 | germplasm strain | 4 | 0.046 | 1186 | niutougou | Local varieties | 1 | -0.057 |
| 408 | daqingmiao | Local varieties | 5 | -0.185 | 1187 | chigu6hao | Local varieties | 2 | -0.143 |
| 409 | hongzhangu | Local varieties | 5 | -0.445 | 1188 | 155-43 | germplasm strain | 5 | -0.009 |
| 410 | hongmiao | Local varieties | 5 | -0.408 | 1189 | maomaogu | Local varieties | 5 | 0.086 |
| 411 | daobaqi | Local varieties | 5 | -0.251 | 1190 | xiaobaimi | Local varieties | 5 | 0.001 |
| 412 | shengtougu | Local varieties | 5 | -0.168 | 1191 | shengzijin | Local varieties | 5 | -0.067 |
| 413 | dahongmiao | Local varieties | 5 | -0.066 | 1192 | 164-14 | germplasm strain | 3 | 0.105 |
| 414 | huangshagu | Local varieties | 5 | 0.058 | 1193 | liulengzi | Local varieties | 3 | 0.075 |
| 415 | zaopi | Local varieties | 5 | -0.274 | 1194 | shengzitou | Local varieties | 5 | 0.094 |
| 416 | huangshagu | Local varieties | 5 | -0.126 | 1195 | erbaigu | Local varieties | 4 | 0.393 |
| 417 | dangdizhong | Local varieties | 2 | -0.357 | 1196 | laohuwei | Local varieties | 5 | -0.034 |
| 418 | gougenhong | Local varieties | 5 | -0.032 | 1197 | dabaigu | Local varieties | 3 | -0.109 |
| 419 | maozhuazi | Local varieties | 5 | 0.041 | 1198 | daobaqi | Local varieties | 3 | -0.068 |
| 420 | huangshazierhao | Local varieties | 5 | -0.260 | 1199 | liulengzi | Local varieties | 5 | 0.352 |
| 421 | qianchuanzi | Local varieties | 5 | -0.016 | 1200 | danaodai | Local varieties | 3 | 0.509 |
| 422 | daobaqi | Local varieties | 5 | -0.296 | 1201 | yapoche | Local varieties | 5 | -0.005 |
| 423 | baishagu | Local varieties | 5 | -0.169 | 1202 | hadaqing | Local varieties | 5 | 0.340 |
| 424 | huangshagu | Local varieties | 5 | -0.047 | 1203 | xiaolibai | Local varieties | 1 | 0.068 |
| 425 | daobaqi | Local varieties | 5 | -0.117 | 1204 | zhongzagu64 | selected variety | 4 | -0.002 |
| 426 | yazizui | Local varieties | 5 | -0.249 | 1205 | zhongzagu90 | selected variety | 3 | 0.157 |
| 427 | zaopiyidaobaqi | Local varieties | 5 | 0.044 | 1206 | zhonggu855 | selected variety | 1 | -0.406 |
| 428 | huangshagu | Local varieties | 5 | -0.324 | 1207 | zhonggu303 | selected variety | 1 | -0.130 |
| 429 | huangzhangu | Local varieties | 5 | -0.184 | 1208 | jinmiaoK1 | selected variety | 4 | 0.037 |
| 430 | huangzhangu | Local varieties | 5 | -0.198 | 1209 | jinmiaoK4 | selected variety | 4 | 0.123 |
| 431 | yugu | Local varieties | 5 | -0.398 | 1210 | jinmiaoK7 | selected variety | 4 | 0.424 |
| 432 | yapoche | Local varieties | 5 | -0.194 | 1211 | jinmiaoK8 | selected variety | 4 | 0.241 |
| 433 | ganjianhuang | Local varieties | 5 | -0.054 | 1212 | chiguK4 | selected variety | 1 | -0.116 |
| 434 | zaopi | Local varieties | 5 | 0.179 | 1213 | chiguK6 | selected variety | 2 | 0.078 |
| 435 | zaopi | Local varieties | 5 | 0.157 | 1214 | jizajinmiao5hao | selected variety | 1 | -0.149 |
| 436 | dalihuang | Local varieties | 4 | 0.119 | 1215 | jizajinmiao6hao | selected variety | 5 | 0.111 |
| 437 | dabaisha | Local varieties | 4 | 0.107 | 1216 | zhangzagu10 | selected variety | 2 | -0.161 |
| 438 | dabaisha | Local varieties | 5 | -0.074 | 1217 | zhangzagu13 | selected variety | 5 | 0.216 |
| 439 | xiaojinmiao | Local varieties | 5 | -0.165 | 1218 | zhangzagu27 | selected variety | 3 | 0.156 |
| 440 | huangkougen | Local varieties | 5 | -0.083 | 1219 | zhangzagu29 | selected variety | 3 | 0.049 |
| 441 | wannianchen | Local varieties | 5 | 0.205 | 1220 | jinfen121 | selected variety | 3 | 0.110 |
| 442 | hongmiaozaoguzi | Local varieties | 3 | 0.038 | 1221 | hengzao17hao | selected variety | 1 | -0.166 |
| 443 | qingmiaozaoguzi | Local varieties | 5 | -0.081 | 1222 | taixuangu28 | selected variety | 1 | -0.026 |
| 444 | qingmiaozaoguzi | Local varieties | 5 | -0.106 | 1223 | taixuangu35 | selected variety | 1 | -0.252 |
| 445 | baomihun | Local varieties | 5 | -0.050 | 1224 | taixuangu43 | selected variety | 1 | -0.105 |
| 446 | zaohuanggu | Local varieties | 2 | 0.140 | 1225 | chaogu26 | selected variety | 3 | 0.126 |
| 447 | zhangbo | Local varieties | 5 | 0.033 | 1226 | chaozagu2 | selected variety | 4 | 0.124 |
| 448 | jiugenqing | Local varieties | 5 | -0.221 | 1227 | jizagu8hao | selected variety | 4 | 0.211 |
| 449 | xiaojinmiao | Local varieties | 4 | 0.072 | 1228 | jiugu45 | selected variety | 1 | -0.138 |
| 450 | laotoubei | Local varieties | 2 | -0.124 | 1229 | jigu32 | selected variety | 1 | -0.158 |
| 451 | baishagu | Local varieties | 1 | -0.058 | 1230 | menggu7047 | selected variety | 1 | -0.601 |
| 452 | zaopi | Local varieties | 1 | -0.468 | 1231 | baogu928 | selected variety | 1 | -0.178 |
| 453 | daobaqi | Local varieties | 2 | -0.282 | 1232 | yugu6hao | selected variety | 2 | 0.015 |
| 454 | laotoubei | Local varieties | 4 | -0.045 | 1233 | zhenggu678 | selected variety | 1 | -0.258 |
| 455 | kougen | Local varieties | 5 | -0.329 | 1234 | huazaH22-10 | selected variety | 2 | 0.258 |
| 456 | gaohongmiao | Local varieties | 5 | -0.138 | 1235 | longgu66 | selected variety | 5 | -0.137 |
| 457 | 228-33 | germplasm strain | 4 | 0.097 | 1236 | longgu23hao | selected variety | 1 | -0.382 |
| 458 | dabaigu | Local varieties | 1 | -0.383 | 1237 | xingu2hao | selected variety | 3 | 0.133 |
| 459 | zhiboting | Local varieties | 1 | -0.008 | 1238 | nenxuan20 | selected variety | 1 | -0.032 |
| 460 | hongmiaogu | Local varieties | 5 | 0.294 | 1239 | yangu14 | selected variety | 1 | -0.174 |
| 461 | bailuguzi | Local varieties | 5 | 0.035 | 1240 | jingu6hao | selected variety | 4 | -0.200 |
| 462 | kangulao | Local varieties | 5 | -0.115 | 1241 | longgu13 | selected variety | 3 | -0.014 |
| 463 | huangyouziweiba | Local varieties | 5 | -0.321 | 1242 | long029 | selected variety | 3 | 0.035 |
| 464 | daobaqi | Local varieties | 2 | -0.099 | 1243 | dungu1hao | selected variety | 4 | -0.120 |
| 465 | dalihuang | Local varieties | 2 | -0.029 | 1244 | jiugu23 | selected variety | 1 | 0.068 |
| 466 | shuilizhan | Local varieties | 5 | 0.345 | 1245 | zhonggu9hao | selected variety | 1 | 0.069 |
| 467 | langmaoqing | Local varieties | 5 | 0.286 | 1246 | zhangnong35 | selected variety | 1 | -0.438 |
| 468 | biansanbian | Local varieties | 3 | 0.177 | 1247 | jingu21 | selected variety | 5 | -0.398 |
| 469 | biandihong | Local varieties | 5 | 0.430 | 1248 | zhongliang15 | selected variety | 5 | 0.196 |
| 470 | hailihong | Local varieties | 5 | -0.038 | 1249 | zhonggu2hao | selected variety | 1 | -0.068 |
| 471 | gaidihong | Local varieties | 5 | -0.213 | 1250 | jigu41 | selected variety | 1 | 0.020 |
| 472 | laohuwei | Local varieties | 4 | -0.006 | 1251 | longgu25 | selected variety | 5 | 0.341 |
| 473 | dabaisha | Local varieties | 4 | -0.059 | 1252 | datong29 | selected variety | 3 | 0.176 |
| 474 | dabaisha | Local varieties | 4 | -0.146 | 1253 | shanxihonggu | selected variety | 5 | 0.197 |
| 475 | dajinmiao | Local varieties | 2 | 0.121 | 1254 | zhangnong47 | selected variety | 1 | -0.172 |
| 476 | datouhuang | Local varieties | 2 | -0.043 | 1255 | longgu13 | selected variety | 1 | -0.424 |
| 477 | hongmiaoqianjinzhui | Local varieties | 5 | 0.062 | 1256 | nenxuan18 | selected variety | 3 | -0.043 |
| 478 | baiganbaisha | Local varieties | 2 | -0.062 | 1257 | longgu38 | selected variety | 5 | 0.064 |
| 479 | shandongbaisha | Local varieties | 5 | 0.431 | 1258 | chaogu58 | selected variety | 1 | -0.394 |
| 480 | huangmiaohuang | Local varieties | 4 | 0.129 | 1259 | nenxuan18 | selected variety | 5 | -0.060 |
| 481 | shandonghuang | Local varieties | 5 | 0.288 | 1260 | zhangza16 | selected variety | 5 | 0.012 |
| 482 | huangqitou | Local varieties | 5 | 0.397 | 1261 | zhangnong47 | selected variety | 1 | 0.056 |
| 483 | xiaoguogu | Local varieties | 5 | -0.037 | 1262 | shanxihonggu | selected variety | 1 | -0.241 |
| 484 | laolaibian | Local varieties | 5 | 0.198 | 1263 | longgu13 | selected variety | 3 | 0.174 |
| 485 | kougen | Local varieties | 5 | 0.322 | 1264 | chaogu58 | selected variety | 1 | -0.342 |
| 486 | hongmiao | Local varieties | 5 | 0.171 | 1265 | zhangza13 | selected variety | 3 | -0.133 |
| 487 | hongmiaoguzi | Local varieties | 5 | 0.205 | 1266 | datong29 | selected variety | 1 | -0.059 |
| 488 | hongqitou | Local varieties | 5 | 0.206 | 1267 | zhangnong35 | selected variety | 4 | -0.194 |
| 489 | liuleng | Local varieties | 5 | -0.191 | 1268 | longgu38 | selected variety | 4 | -0.052 |
| 490 | dalihuang | Local varieties | 1 | 0.242 | 1269 | zhonggu2hao | selected variety | 4 | -0.104 |
| 491 | laotoubei | Local varieties | 1 | -0.117 | 1270 | longgu25 | selected variety | 1 | -0.166 |
| 492 | huangzhangu | Local varieties | 1 | -0.319 | 1271 | nenxuan15 | selected variety | 1 | -0.073 |
| 493 | shengjinbocaigen | Local varieties | 1 | -0.008 | 1272 | longgu38 | selected variety | 3 | -0.042 |
| 494 | shuihonggen | Local varieties | 2 | 0.006 | 1273 | zhonggu2hao | selected variety | 3 | 0.365 |
| 495 | dalihuang | Local varieties | 2 | -0.131 | 1274 | chaogu58 | selected variety | 5 | -0.278 |
| 496 | danaohuang | Local varieties | 1 | -0.229 | 1275 | jinmiaoK2 | selected variety | 4 | -0.488 |
| 497 | dalihuang | Local varieties | 1 | -0.087 | 1276 | gongai88 | selected variety | 4 | -0.267 |
| 498 | qianchuanzi | Local varieties | 1 | 0.042 | 1277 | longgu125 | selected variety | 3 | -0.088 |
| 499 | yapoche | Local varieties | 5 | 0.054 | 1278 | jigu168 | selected variety | 1 | -0.165 |
| 500 | qingmiao | Local varieties | 5 | 0.075 | 1279 | jigu39 | selected variety | 1 | -0.400 |
| 501 | dajinmiao | Local varieties | 3 | -0.078 | 1280 | zhonggu9hao | selected variety | 5 | -0.003 |
| 502 | xiaobocaigen | Local varieties | 5 | 0.138 | 1281 | nenxuan18 | selected variety | 3 | 0.060 |
| 503 | wazijiao | Local varieties | 4 | 0.336 | 1282 | zhangnong47 | selected variety | 1 | -0.326 |
| 504 | huliwei | Local varieties | 5 | 0.423 | 1283 | longgu13 | selected variety | 3 | -0.036 |
| 505 | jinsui | Local varieties | 5 | 0.298 | 1284 | nenxuan15 | selected variety | 5 | 0.278 |
| 506 | laolaibian | Local varieties | 4 | 0.101 | 1285 | shanxihonggu | selected variety | 5 | 0.046 |
| 507 | laolaibian | Local varieties | 5 | 0.088 | 1286 | zhangza16 | selected variety | 4 | -0.211 |
| 508 | baodigao | Local varieties | 5 | 0.201 | 1287 | zhangnong35 | selected variety | 4 | -0.071 |
| 509 | gaizhouhong | Local varieties | 3 | 0.174 | 1288 | chiyoujinmiao1hao | selected variety | 4 | 0.073 |
| 510 | yazizui | Local varieties | 4 | -0.065 | 1289 | datong29 | selected variety | 1 | -0.068 |
| 511 | datouhuang | Local varieties | 4 | 0.266 | 1290 | 178-16 2016 | germplasm strain | 4 | 0.031 |
| 512 | daobaqi | Local varieties | 5 | -0.061 | 1291 | 178-6 | germplasm strain | 4 | -0.004 |
| 513 | hongzhangu | Local varieties | 4 | 0.253 | 1292 | huanggu13 | selected variety | 1 | -0.065 |
| 514 | jinxiangyu | Local varieties | 4 | 0.108 | 1293 | 154-24 | germplasm strain | 4 | 0.042 |
| 515 | bailucheng | Local varieties | 4 | 0.044 | 1294 | shenggu1hao | selected variety | 3 | -0.422 |
| 516 | laolaibian | Local varieties | 4 | 0.124 | 1295 | jiu16×317-6 | germplasm strain | 3 | -0.382 |
| 517 | daobaqi | Local varieties | 5 | -0.054 | 1296 | 166-92-17 | germplasm strain | 1 | -0.137 |
| 518 | daihuanggu | Local varieties | 3 | 0.025 | 1297 | 166-92-18 | germplasm strain | 3 | -0.074 |
| 519 | yapoche | Local varieties | 2 | -0.116 | 1298 | 166-92-19 | germplasm strain | 5 | -0.006 |
| 520 | gaowafan | Local varieties | 5 | 0.219 | 1299 | 12-19-20 | germplasm strain | 3 | 0.172 |
| 521 | taihuang | Local varieties | 5 | 0.053 | 1300 | 17-2 | germplasm strain | 3 | 0.294 |
| 522 | daobaqi | Local varieties | 5 | -0.079 | 1301 | 16-28-24 | germplasm strain | 5 | 0.056 |
| 523 | daobaqi | Local varieties | 5 | 0.106 | 1302 | 16-28-25 | germplasm strain | 5 | -0.013 |
| 524 | longhuangsan | Local varieties | 5 | -0.181 | 1303 | 16-28-26 | germplasm strain | 5 | 0.037 |
| 525 | dalihuang | Local varieties | 1 | -0.201 | 1304 | 16-28-27 | germplasm strain | 5 | 0.152 |
| 526 | yangqiangdui | Local varieties | 4 | 0.269 | 1305 | 16-59-28 | germplasm strain | 3 | 0.030 |
| 527 | laohuwei | Local varieties | 3 | 0.179 | 1306 | 16-59-29 | germplasm strain | 5 | 0.520 |
| 528 | daqingmiao | Local varieties | 5 | 0.283 | 1307 | 16-59-30 | germplasm strain | 3 | 0.031 |
| 529 | yazizui | Local varieties | 2 | -0.066 | 1308 | 18-6-31 | germplasm strain | 4 | -0.077 |
| 530 | longfengsan | Local varieties | 5 | 0.204 | 1309 | 18-6-32 | germplasm strain | 4 | 0.073 |
| 531 | laolaibian | Local varieties | 1 | -0.317 | 1310 | 18-6-33 | germplasm strain | 4 | 0.186 |
| 532 | daihuanggu | Local varieties | 4 | 0.091 | 1311 | 18-6-34 | germplasm strain | 4 | 0.443 |
| 533 | hongganbai | Local varieties | 5 | 0.247 | 1312 | 18-6-35 | germplasm strain | 3 | 0.220 |
| 534 | datouhuang | Local varieties | 4 | -0.128 | 1313 | 18-6-36 | germplasm strain | 4 | 0.180 |
| 535 | gaowazao | Local varieties | 5 | -0.046 | 1314 | 18-6-38 | germplasm strain | 4 | 0.308 |
| 536 | heibohan | Local varieties | 4 | 0.173 | 1315 | 18-6-39 | germplasm strain | 3 | 0.223 |
| 537 | baodigao | Local varieties | 5 | 0.154 | 1316 | 18-6-40 | germplasm strain | 4 | -0.135 |
| 538 | jinmiao | Local varieties | 2 | -0.008 | 1317 | 18-6-41 | germplasm strain | 3 | 0.118 |
| 539 | dalihuang | Local varieties | 4 | 0.022 | 1318 | 20-44-43 | germplasm strain | 4 | -0.014 |
| 540 | baodigaozhangu | Local varieties | 1 | -0.188 | 1319 | 20-44-44 | germplasm strain | 3 | -0.062 |
| 541 | laohuwei | Local varieties | 5 | 0.092 | 1320 | 21-1-45 | germplasm strain | 3 | 0.242 |
| 542 | baiguzi | Local varieties | 5 | -0.011 | 1321 | 21-1-48 | germplasm strain | 4 | 0.086 |
| 543 | liulengzi | Local varieties | 5 | -0.230 | 1322 | 21-1-49 | germplasm strain | 3 | -0.035 |
| 544 | esiniu | Local varieties | 5 | -0.037 | 1323 | 21-1-50 | germplasm strain | 4 | 0.054 |
| 545 | qisifeng | Local varieties | 5 | -0.110 | 1324 | 21-1-51 | germplasm strain | 4 | 0.104 |
| 546 | dabaigu | Local varieties | 2 | -0.019 | 1325 | 21-1-52 | germplasm strain | 3 | 0.016 |
| 547 | zuanzitou | Local varieties | 3 | -0.293 | 1326 | 24-17-53 | germplasm strain | 4 | 0.001 |
| 548 | xiaoliulengzi | Local varieties | 5 | -0.127 | 1327 | 24-17-54 | germplasm strain | 4 | 0.176 |
| 549 | qingmiaozuanzitou | Local varieties | 5 | -0.021 | 1328 | 24-17-55 | germplasm strain | 3 | -0.012 |
| 550 | zuanzitou | Local varieties | 5 | -0.010 | 1329 | 24-17-56 | germplasm strain | 5 | 0.255 |
| 551 | chengpotun | Local varieties | 3 | -0.435 | 1330 | 28-54 | germplasm strain | 4 | 0.606 |
| 552 | shengzijin | Local varieties | 5 | -0.282 | 1331 | 28-57 | germplasm strain | 4 | 0.262 |
| 553 | xiaobacha | Local varieties | 5 | -0.360 | 1332 | 26-3-59 | germplasm strain | 4 | -0.060 |
| 554 | liushitianhaicang | Local varieties | 1 | -0.044 | 1333 | 26-3-60 | germplasm strain | 4 | -0.017 |
| 555 | shengzijin | Local varieties | 5 | 0.030 | 1334 | 26-3-61 | germplasm strain | 4 | 0.161 |
| 556 | tiepazi | Local varieties | 5 | 0.026 | 1335 | 26-3-62 | germplasm strain | 3 | 0.154 |
| 557 | niubozi | Local varieties | 2 | -0.598 | 1336 | 26-3-63 | germplasm strain | 4 | 0.195 |
| 558 | baizuanzitou | Local varieties | 5 | 0.125 | 1337 | 26-3-64 | germplasm strain | 4 | 0.351 |
| 559 | zuanzitou | Local varieties | 5 | -0.228 | 1338 | 26-3-65 | germplasm strain | 4 | 0.196 |
| 560 | ganjianzixiaobaigu | Local varieties | 2 | -0.251 | 1339 | 26-3-66 | germplasm strain | 4 | 0.260 |
| 561 | qingmiaoganjianzi | Local varieties | 2 | -0.213 | 1340 | YN-67 | germplasm strain | 3 | 0.141 |
| 562 | jiantouxiaobaigu | Local varieties | 1 | -0.355 | 1341 | YN-68 | germplasm strain | 3 | 0.148 |
| 563 | xiaoqingmiao | Local varieties | 5 | 0.083 | 1342 | YN-70 | germplasm strain | 3 | 0.195 |
| 564 | 228-16 | germplasm strain | 4 | 0.118 | 1343 | YN-71 | germplasm strain | 3 | 0.169 |
| 565 | bagoudao | Local varieties | 3 | 0.071 | 1344 | YN-73 | germplasm strain | 4 | 0.146 |
| 566 | ganjianzi | Local varieties | 2 | 0.199 | 1345 | YN-74 | germplasm strain | 3 | -0.104 |
| 567 | zhuyeqing | Local varieties | 5 | -0.105 | 1346 | YN-75 | germplasm strain | 3 | 0.027 |
| 568 | dugentou | Local varieties | 5 | -0.159 | 1347 | YN-76 | germplasm strain | 3 | 0.058 |
| 569 | bosuogeda | Local varieties | 5 | -0.048 | 1348 | YN-77 | germplasm strain | 5 | -0.001 |
| 570 | liushitianhaicang | Local varieties | 3 | -0.405 | 1349 | YN-78 | germplasm strain | 3 | 0.170 |
| 571 | niutougou | Local varieties | 2 | 0.059 | 1350 | YN-79 | germplasm strain | 4 | 0.181 |
| 572 | liulengzi | Local varieties | 2 | 0.043 | 1351 | YN-81 | germplasm strain | 4 | 0.335 |
| 573 | hongmiaobangzishu | Local varieties | 5 | -0.252 | 1352 | YN-82 | germplasm strain | 4 | 0.153 |
| 574 | hongmiaohuapibai | Local varieties | 2 | -0.475 | 1353 | YN-83 | germplasm strain | 2 | 0.096 |
| 575 | mangzhongbai | Local varieties | 3 | 0.203 | 1354 | YN-85 | germplasm strain | 3 | 0.319 |
| 576 | xiaohuashubaigu | Local varieties | 2 | -0.509 | 1355 | YN-86 | germplasm strain | 3 | 0.076 |
| 577 | yuci | Local varieties | 3 | -0.057 | 1356 | YN-87 | germplasm strain | 4 | 0.169 |
| 578 | ganjianzihuangmi | Local varieties | 5 | -0.161 | 1357 | YN-88 | germplasm strain | 4 | 0.184 |
| 579 | qingmiaohuapibai | Local varieties | 3 | -0.302 | 1358 | YN-89 | germplasm strain | 3 | 0.029 |
| 580 | dabaiguxiaobaimi | Local varieties | 3 | -0.027 | 1359 | YN-90 | germplasm strain | 3 | 0.200 |
| 581 | bocaigen | Local varieties | 5 | -0.051 | 1360 | YN-91 | germplasm strain | 4 | -0.082 |
| 582 | lamahuang | Local varieties | 5 | -0.003 | 1361 | YN-92 | germplasm strain | 4 | -0.173 |
| 583 | xiaojinmiao | Local varieties | 5 | -0.146 | 1362 | YN-93 | germplasm strain | 4 | 0.044 |
| 584 | liushitianhaicang | Local varieties | 3 | 0.043 | 1363 | YN-94 | germplasm strain | 4 | 0.071 |
| 585 | hongmiaoxiaobaimi | Local varieties | 5 | -0.152 | 1364 | YN-95 | germplasm strain | 4 | 0.475 |
| 586 | hongmiaozijianzi | Local varieties | 5 | -0.345 | 1365 | YN-97 | germplasm strain | 4 | 0.463 |
| 587 | kaoshanhong | Local varieties | 5 | 0.094 | 1366 | YN-98 | germplasm strain | 3 | 0.120 |
| 588 | hongmiaozuanzitou | Local varieties | 5 | -0.229 | 1367 | YN-99 | germplasm strain | 3 | 0.168 |
| 589 | yapoche | Local varieties | 5 | 0.145 | 1368 | YN-100 | germplasm strain | 3 | 0.091 |
| 590 | hongmiaomaozhao | Local varieties | 5 | 0.056 | 1369 | YN-101 | germplasm strain | 3 | 0.117 |
| 591 | xiaohongguzi | Local varieties | 5 | 0.159 | 1370 | YN-102 | germplasm strain | 3 | 0.215 |
| 592 | lamahuang | Local varieties | 5 | 0.252 | 1371 | YN-103 | germplasm strain | 3 | 0.126 |
| 593 | hongmiaohuashubai | Local varieties | 3 | -0.202 | 1372 | YN-104 | germplasm strain | 3 | 0.248 |
| 594 | xiaobaigu | Local varieties | 5 | 0.409 | 1373 | YN-105 | germplasm strain | 3 | 0.286 |
| 595 | hongmiaozuanzitou | Local varieties | 5 | -0.094 | 1374 | YN-107 | germplasm strain | 4 | 0.208 |
| 596 | hongmiaohuapibai | Local varieties | 3 | 0.086 | 1375 | YN-108 | germplasm strain | 3 | -0.022 |
| 597 | gegegu | Local varieties | 5 | 0.145 | 1376 | YN-109 | germplasm strain | 3 | -0.021 |
| 598 | hongmiaohuapibai | Local varieties | 5 | 0.298 | 1377 | YN-110 | germplasm strain | 4 | 0.075 |
| 599 | zhangboyan | Local varieties | 5 | 0.161 | 1378 | YN-111 | germplasm strain | 3 | 0.088 |
| 600 | huapibai | Local varieties | 5 | -0.232 | 1379 | YN-112 | germplasm strain | 3 | 0.013 |
| 601 | qingmiaoshanxibai | Local varieties | 5 | -0.192 | 1380 | YN-113 | germplasm strain | 3 | 0.229 |
| 602 | xiaobacha | Local varieties | 5 | 0.054 | 1381 | YN-114 | germplasm strain | 3 | 0.030 |
| 603 | xiaoqingmiao | Local varieties | 5 | 0.350 | 1382 | YN-117 | germplasm strain | 5 | 0.112 |
| 604 | guandongqing | Local varieties | 5 | 0.252 | 1383 | YN-118 | germplasm strain | 3 | 0.120 |
| 605 | qingmiaoshengtoujin | Local varieties | 5 | -0.220 | 1384 | YN-119 | germplasm strain | 3 | 0.342 |
| 606 | baimaomaogu | Local varieties | 5 | -0.051 | 1385 | YN-120 | germplasm strain | 3 | 0.094 |
| 607 | huiningmixiahonggu | Local varieties | 5 | 0.091 | 1386 | YN-121 | germplasm strain | 4 | 0.008 |
| 608 | pingliangmaocaogu | Local varieties | 5 | 0.182 | 1387 | YN-122 | germplasm strain | 4 | 0.266 |
| 609 | niumaohuang | Local varieties | 4 | -0.059 | 1388 | YN-123 | germplasm strain | 4 | 0.152 |
| 610 | xiaohonggu | Local varieties | 5 | 0.126 | 1389 | YN-124 | germplasm strain | 4 | 0.097 |
| 611 | dingxidaheicaogu | Local varieties | 5 | -0.177 | 1390 | YN-125 | germplasm strain | 3 | -0.003 |
| 612 | heimaogu | Local varieties | 5 | 0.296 | 1391 | YN-126 | germplasm strain | 3 | -0.143 |
| 613 | xiaomaolianggu | Local varieties | 5 | -0.275 | 1392 | YN-127 | germplasm strain | 4 | -0.119 |
| 614 | gaolanjiatougu | Local varieties | 5 | 0.014 | 1393 | YN-128 | germplasm strain | 3 | 0.087 |
| 615 | jintahongmaogu | Local varieties | 5 | 0.219 | 1394 | YN-129 | germplasm strain | 4 | -0.003 |
| 616 | zhangyehuangdagu | Local varieties | 3 | 0.095 | 1395 | YN-130 | germplasm strain | 4 | 0.042 |
| 617 | zhangyeanzhenhuanggu | Local varieties | 5 | 0.599 | 1396 | 28-35 | germplasm strain | 4 | 0.335 |
| 618 | xiaobaishagu | Local varieties | 5 | -0.196 | 1397 | 28-9 | germplasm strain | 4 | 0.606 |
| 619 | yongjingxiaoshazhou | Local varieties | 5 | -0.358 | 1398 | 28-47 | germplasm strain | 3 | 0.150 |
| 620 | dagukou | Local varieties | 3 | 0.293 | 1399 | 30-27 | germplasm strain | 3 | -0.190 |
| 621 | baicaohonggu | Local varieties | 5 | -0.002 | 1400 | 30-8 | germplasm strain | 1 | -0.274 |
| 622 | dongxianghongmaogu | Local varieties | 5 | 0.501 | 1401 | 37-1 | germplasm strain | 4 | 0.088 |
| 623 | tongweixiaohuanggu | Local varieties | 5 | -0.018 | 1402 | 176 | germplasm strain | 4 | -0.015 |
| 624 | tongweixiaozhaogu | Local varieties | 5 | -0.280 | 1403 | 174-7 | germplasm strain | 4 | 0.127 |
| 625 | longxixiaojinhuang | Local varieties | 5 | 0.120 | 1404 | 176-2 | germplasm strain | 4 | 0.226 |
| 626 | yongdengmihuanggu | Local varieties | 3 | -0.214 | 1405 | 176 | germplasm strain | 4 | 0.415 |
| 627 | dingxixiaozhuyeqing | Local varieties | 2 | 0.152 | 1406 | 176 | germplasm strain | 4 | 0.196 |
| 628 | zigansihao | Local varieties | 5 | 0.375 | 1407 | 176 | germplasm strain | 4 | 0.025 |
| 629 | danxuanxibaihui | Local varieties | 5 | 0.028 | 1408 | 69-10 | germplasm strain | 3 | 0.344 |
| 630 | huiningheigu | Local varieties | 5 | -0.593 | 1409 | 5-20 | germplasm strain | 3 | 0.044 |
| 631 | dingxidabaigu | Local varieties | 5 | 0.034 | 1410 | 5-20 | germplasm strain | 3 | 0.042 |
| 632 | zuanzihuanggu | Local varieties | 5 | 0.097 | 1411 | 5-27 | germplasm strain | 5 | -0.106 |
| 633 | ganniubian | Local varieties | 5 | -0.073 | 1412 | 5-27 | germplasm strain | 5 | 0.124 |
| 634 | dingxihongcaogu | Local varieties | 5 | 0.264 | 1413 | long14-7014 | germplasm strain | 2 | -0.281 |
| 635 | daheigu | Local varieties | 5 | 0.206 | 1414 | jiugu11 | selected variety | 3 | -0.046 |
| 636 | daheigu | Local varieties | 2 | 0.066 | 1415 | JM002 | germplasm strain | 1 | -0.055 |
| 637 | wuweihuangmaogu | Local varieties | 2 | 0.244 | 1416 | K0921 | germplasm strain | 3 | -0.100 |
| 638 | 228-38 | germplasm strain | 4 | 0.036 | 1417 | F2bei | germplasm strain | 3 | -0.315 |
| 639 | yongchangxiaobaigu | Local varieties | 5 | 0.526 | 1418 | 101-2 | germplasm strain | 3 | 0.446 |
| 640 | langweiba | Local varieties | 5 | 0.113 | 1419 | 4-5 | germplasm strain | 2 | 0.084 |
| 641 | laohuwei | Local varieties | 5 | 0.088 | 1420 | K0921 | germplasm strain | 3 | 0.007 |
| 642 | shengtoujin | Local varieties | 5 | 0.263 | 1421 | K0921 | germplasm strain | 3 | 0.050 |
| 643 | ganlierhao | Local varieties | 2 | 0.256 | 1422 | menglonghongmiaohonggu | Local varieties | 5 | 0.220 |
| 644 | gaolandengziqi | Local varieties | 2 | 0.133 | 1423 | menglongmaomaogu | Local varieties | 3 | 0.352 |
| 645 | gaolanmihuanggu | Local varieties | 2 | 0.078 | 1424 | 151-45 | germplasm strain | 3 | 0.162 |
| 646 | dingxiqingshuigu | Local varieties | 5 | 0.234 | 1425 | 153-26 | germplasm strain | 5 | -0.271 |
| 647 | yapoche | Local varieties | 3 | -0.230 | 1426 | 153-31 | germplasm strain | 5 | 0.050 |
| 648 | erbaigu | Local varieties | 5 | 0.120 | 1427 | 153-35 | germplasm strain | 4 | -0.159 |
| 649 | daihuanggu | Local varieties | 5 | 0.131 | 1428 | 154-25 | germplasm strain | 1 | -0.127 |
| 650 | baijingu | Local varieties | 3 | -0.238 | 1429 | 341 | germplasm strain | 3 | -0.168 |
| 651 | gaolandaihuanggu | Local varieties | 5 | 0.539 | 1430 | 627 | germplasm strain | 3 | -0.264 |
| 652 | gaolanshenchuanshazhougu | Local varieties | 5 | 0.134 | 1431 | 687 | germplasm strain | 4 | 0.262 |
| 653 | dingxishazhougu | Local varieties | 5 | 0.324 | 1432 | 162-2 | germplasm strain | 5 | 0.597 |
| 654 | huininglvjiangsheng | Local varieties | 3 | 0.390 | 1433 | 50-6 | germplasm strain | 1 | 0.112 |
| 655 | jiuquanjinwohuanggu | Local varieties | 5 | 0.543 | 1434 | 50-10 | germplasm strain | 3 | -0.090 |
| 656 | baiganqixian | Local varieties | 5 | 0.284 | 1435 | chiyou13-8 | germplasm strain | 2 | -0.600 |
| 657 | hongxianqi | Local varieties | 5 | 0.051 | 1436 | 180-3 | germplasm strain | 5 | 0.535 |
| 658 | erqixian | Local varieties | 5 | 0.101 | 1437 | chi1623 | germplasm strain | 5 | 0.130 |
| 659 | bayuehuang | Local varieties | 3 | -0.038 | 1438 | chi158-17 | germplasm strain | 4 | -0.234 |
| 660 | mujiju | Local varieties | 3 | 0.399 | 1439 | 49-41 | germplasm strain | 4 | 0.081 |
| 661 | esilv | Local varieties | 3 | 0.083 | 1440 | 162-21 | germplasm strain | 3 | 0.275 |
| 662 | xiaohonggu | Local varieties | 3 | 0.298 | 1441 | 141-44 | germplasm strain | 4 | -0.080 |
| 663 | jiuguzi | Local varieties | 1 | -0.065 | 1442 | fenghonggu | Local varieties | 5 | -0.084 |
| 664 | heiguzi | Local varieties | 5 | 0.016 | 1443 | 201-76 | germplasm strain | 5 | 0.021 |
| 665 | langweiba | Local varieties | 5 | 0.252 | 1444 | 27-33 | germplasm strain | 5 | 0.552 |
| 666 | xiaohuanggu | Local varieties | 3 | 0.432 | 1445 | chigu5hao | selected variety | 5 | 0.180 |
| 667 | jiugenqi | Local varieties | 3 | 0.032 | 1446 | chigu6hao | selected variety | 5 | -0.079 |
| 668 | laobaigu | Local varieties | 3 | 0.137 | 1447 | chigu7hao | selected variety | 5 | 0.387 |
| 669 | xiaohuanggu | Local varieties | 5 | 0.269 | 1448 | chigu8hao | selected variety | 5 | 0.141 |
| 670 | honggenjiugu | Local varieties | 3 | 0.200 | 1449 | chigu9hao | selected variety | 5 | 0.153 |
| 671 | lanlaopo | Local varieties | 1 | -0.036 | 1450 | chigu10hao | selected variety | 3 | 0.124 |
| 672 | hongjiugu | Local varieties | 5 | 0.126 | 1451 | fenggu11 | selected variety | 3 | 0.321 |
| 673 | qigenqi | Local varieties | 3 | -0.292 | 1452 | chigu16 | selected variety | 3 | 0.148 |
| 674 | bailongzhaojiugu | Local varieties | 3 | 0.025 | 1453 | chigu17 | selected variety | 3 | 0.161 |
| 675 | daihuanggu | Local varieties | 3 | 0.254 | 1454 | chigu18 | selected variety | 3 | -0.009 |
| 676 | dayuanjinxian | Local varieties | 3 | 0.273 | 1455 | menggu6hao | selected variety | 5 | -0.228 |
| 677 | xiaohuanggu | Local varieties | 3 | 0.116 | 1456 | menggu6hao | selected variety | 5 | -0.342 |
| 678 | baikagu | Local varieties | 3 | 0.059 | 1457 | zhaonong21 | selected variety | 3 | 0.264 |
| 679 | shekougu | Local varieties | 3 | 0.156 | 1458 | chaonongdabaigu | Local varieties | 3 | 0.119 |
| 680 | lanlaopo | Local varieties | 2 | -0.224 | 1459 | fenghonggu | Local varieties | 5 | 0.035 |
| 681 | xiaoliaojiao | Local varieties | 3 | -0.096 | 1460 | shanxihonggu | Local varieties | 5 | -0.090 |
| 682 | daqigu | Local varieties | 3 | 0.276 | 1461 | beizifushanxihonggu | Local varieties | 5 | 0.025 |
| 683 | daihuanggu | Local varieties | 3 | 0.284 | 1462 | hongshanqushanxihonggu | Local varieties | 5 | 0.109 |
| 684 | hongguzi | Local varieties | 2 | -0.110 | 1463 | wangfushanxihonggu | Local varieties | 5 | -0.121 |
| 685 | daobaqi | Local varieties | 1 | -0.112 | 1464 | ningchengshanxihonggu | Local varieties | 5 | 0.337 |
| 686 | kagu | Local varieties | 3 | -0.135 | 1465 | xiqiaoshanxihonggu | Local varieties | 5 | 0.125 |
| 687 | yangu3hao | Local varieties | 1 | 0.053 | 1466 | xiqiaoshanxihonggu | Local varieties | 5 | 0.033 |
| 688 | 228-35 | germplasm strain | 3 | 0.470 | 1467 | songshanqushanxihonggu | Local varieties | 5 | 0.133 |
| 689 | heigaiyi | Local varieties | 3 | 0.141 | 1468 | songshanqushanxihonggu | Local varieties | 5 | 0.078 |
| 690 | xiaoqixian | Local varieties | 5 | 0.346 | 1469 | aohanhonggu | Local varieties | 5 | 0.036 |
| 691 | yatache | Local varieties | 2 | -0.796 | 1470 | zhaoyanghonggu | Local varieties | 5 | 0.117 |
| 692 | kouwaibai | Local varieties | 4 | -0.185 | 1471 | hongkegu | Local varieties | 5 | 0.347 |
| 693 | heiyelai | Local varieties | 2 | -0.194 | 1472 | ai88 | 遗传材料 | 1 | -0.647 |
| 694 | hongliushi | Local varieties | 5 | 0.104 | 1473 | gongai2hao | 遗传材料 | 1 | -0.559 |
| 695 | shandongbaiA | Local varieties | 1 | -0.315 | 1474 | gongai60 | 遗传材料 | 2 | 0.020 |
| 696 | qingkegu | Local varieties | 3 | -0.163 | 1475 | dungu1hao | selected variety | 1 | -0.095 |
| 697 | erhonggu | Local varieties | 3 | 0.340 | 1476 | yugu1hao | selected variety | 1 | -0.181 |
| 698 | baimaolianggu | Local varieties | 3 | 0.344 | 1477 | hangkong8hao | Other germplasm | 2 | -0.927 |
| 699 | longzhaoxiaogu | Local varieties | 3 | -0.013 | 1478 | jiugu11 | selected variety | 2 | -0.888 |
| 700 | xiaoshipaogu | Local varieties | 5 | 0.269 | 1479 | aohannahuixiaobaimi | Local varieties | 5 | -0.029 |
| 701 | hongfuchuan | Local varieties | 1 | -0.174 | 1480 | jinxiangyuxiaobaimi | Local varieties | 5 | 0.307 |
| 702 | bocaitouguzi | Local varieties | 3 | 0.409 | 1481 | jinxiangyuxiaobaimi | Local varieties | 5 | 0.372 |
| 703 | daobaqi | Local varieties | 5 | -0.127 | 1482 | xiaoxiangmi | Local varieties | 1 | -0.398 |
| 704 | longzhaojiugu | Local varieties | 5 | 0.061 | 1483 | yapoche | Local varieties | 5 | 0.261 |
| 705 | erqixian | Local varieties | 3 | 0.348 | 1484 | jingu1hao | selected variety | 1 | -0.162 |
| 706 | huangganxiaohuanggu | Local varieties | 5 | 0.477 | 1485 | jingu2hao | selected variety | 5 | 0.316 |
| 707 | zhufengejiegu | Local varieties | 5 | -0.042 | 1486 | lamahuang | Local varieties | 5 | 0.201 |
| 708 | bayuehuanghonggangu | Local varieties | 3 | 0.331 | 1487 | lvxiaomi | Local varieties | 5 | 0.044 |
| 709 | jiaoni | Local varieties | 3 | 0.041 | 1488 | lvxiaomi | Local varieties | 5 | 0.124 |
| 710 | niumaoxiaohuanggu | Local varieties | 2 | 0.122 | 1489 | yangu1hao | selected variety | 3 | -0.043 |
| 711 | huangguzi | Local varieties | 2 | 0.168 | 1490 | yangu18 | selected variety | 5 | 0.343 |
| 712 | bocaigen | Local varieties | 5 | -0.158 | 1491 | maomaoliang | Local varieties | 3 | 0.337 |
| 713 | gedahuanggu | Local varieties | 3 | 0.034 | 1492 | xiaosuliang | Local varieties | 5 | 0.205 |
| 714 | jinlaiquan | Local varieties | 3 | -0.044 | 1493 | huangqihuang | Local varieties | 5 | -0.110 |
| 715 | jinlaihuang | Local varieties | 3 | 0.268 | 1494 | qinzhouhuang | Local varieties | 2 | -1.069 |
| 716 | jinlaihuang | Local varieties | 3 | 0.027 | 1495 | qinzhouhuang | Local varieties | 2 | -1.049 |
| 717 | bailiusha | Local varieties | 2 | -0.066 | 1496 | longkou2042 | Other germplasm | 3 | 0.023 |
| 718 | dayezhuanhuigu | Local varieties | 2 | 0.053 | 1497 | 2013-327 | germplasm strain | 3 | 0.104 |
| 719 | xiaohuanggu | Local varieties | 2 | -0.127 | 1498 | 2013-341 | germplasm strain | 1 | -0.415 |
| 720 | jiugenqi | Local varieties | 3 | 0.027 | 1499 | heixiaomi | Local varieties | 5 | 0.132 |
| 721 | honggancaogu | Local varieties | 3 | -0.128 | 1500 | heixiaomi | Local varieties | 5 | -0.046 |
| 722 | baiguayin | Local varieties | 3 | 0.156 | 1501 | heikeguzi | Local varieties | 5 | 0.104 |
| 723 | shuangguayin | Local varieties | 3 | 0.379 | 1502 | chaogu2hao | selected variety | 1 | -0.382 |
| 724 | jinguzi | Local varieties | 5 | 0.292 | 1503 | taiyanghuang | Local varieties | 5 | 0.155 |
| 725 | daihuanggu | Local varieties | 3 | 0.000 | 1504 | lvmi | Local varieties | 5 | 0.049 |
| 726 | daihuanggu | Local varieties | 3 | 0.195 | 1505 | jinxiangyu | Local varieties | 5 | 0.189 |
| 727 | huangguzi | Local varieties | 3 | 0.262 | 1506 | yapoche | Local varieties | 5 | 0.438 |
| 728 | huanggu | Local varieties | 5 | 0.505 | 1507 | bocaigen | Local varieties | 5 | 0.739 |
| 729 | qitouhuang | Local varieties | 3 | 0.137 | 1508 | bocaigen | Local varieties | 5 | 0.152 |
| 730 | shengtiaozigu | Local varieties | 3 | -0.009 | 1509 | zhanguhun | Local varieties | 5 | 0.170 |
| 731 | shengshenggu | Local varieties | 3 | 0.474 | 1510 | xiaojinmiao | Local varieties | 4 | 0.262 |
| 732 | qingguzi | Local varieties | 5 | 0.215 | 1511 | jinzhuizi | Local varieties | 5 | 0.148 |
| 733 | bayiyi | Local varieties | 5 | 0.149 | 1512 | maozhuazi | Local varieties | 3 | 0.094 |
| 734 | qiyuehuang | Local varieties | 3 | 0.052 | 1513 | jingu3 | selected variety | 3 | -0.031 |
| 735 | donghuangliang | Local varieties | 3 | 0.023 | 1514 | dongbeinongdaihuanggu | Local varieties | 5 | 0.253 |
| 736 | dadongfangliang | Local varieties | 3 | -0.031 | 1515 | 99ai4Abuyuxi | 遗传材料 | 5 | 0.050 |
| 737 | dongfengliang | Local varieties | 3 | 0.448 | 1516 | huangjingu | Local varieties | 4 | 0.420 |
| 738 | sanbianhua | Local varieties | 3 | -0.020 | 1517 | chutoulang jinmiao | Local varieties | 4 | 0.459 |
| 739 | zhuyeqingⅡ | Local varieties | 3 | 0.300 | 1518 | chutoulang jinmiao | Local varieties | 4 | 0.321 |
| 740 | zhuyeqing | Local varieties | 5 | 0.201 | 1519 | damiao jinmiao | Local varieties | 4 | 0.442 |
| 741 | erhuanggu | Local varieties | 3 | 0.325 | 1520 | dongxinjingdajinmiao | Local varieties | 4 | 0.196 |
| 742 | qianchuanguⅡ | Local varieties | 3 | -0.148 | 1521 | dongxinjingdajinmiao | Local varieties | 4 | 0.425 |
| 743 | bailuhuang | Local varieties | 3 | 0.141 | 1522 | wangfuhuangbacha | Local varieties | 4 | 0.381 |
| 744 | sanganqi | Local varieties | 2 | 0.016 | 1523 | songshanquhuangbacha | Local varieties | 4 | 0.476 |
| 745 | dabairuangu | Local varieties | 3 | -0.047 | 1524 | wengqihuangjingu | Local varieties | 4 | 0.383 |
| 746 | baicaotiehou | Local varieties | 2 | -0.171 | 1525 | aqihuangjingu | Local varieties | 4 | 0.329 |
| 747 | hongqingbian | Local varieties | 3 | -0.012 | 1526 | aohanhuanggu | Local varieties | 4 | 0.058 |
| 748 | haiyagenhong | Local varieties | 3 | 0.155 | 1527 | gulubanhaogailiangdajinmiao | Local varieties | 4 | 0.582 |
| 749 | gehekanzhangmu | Local varieties | 3 | 0.169 | 1528 | niugutuhuangjinmiao | Local varieties | 4 | 0.496 |
| 750 | qiubailu | Local varieties | 3 | 0.070 | 1529 | salibahuangjinmiao | Local varieties | 4 | 0.357 |
| 751 | lixiagu | Local varieties | 3 | 0.339 | 1530 | sidaowanhuangjinmiao | Local varieties | 4 | 0.496 |
| 752 | xiniugu | Local varieties | 3 | 0.391 | 1531 | xinhuihuangjinmiao | Local varieties | 3 | 0.038 |
| 753 | yinshiwugu | Local varieties | 5 | -0.080 | 1532 | manihanhuangjinmiao | Local varieties | 4 | 0.288 |
| 754 | hongyanzhangu | Local varieties | 5 | 0.103 | 1533 | huangjingubufencha | Local varieties | 4 | 0.303 |
| 755 | tieboluo | Local varieties | 3 | -0.248 | 1534 | huangbacha laofu | Local varieties | 4 | 0.433 |
| 756 | xiaoqingping | Local varieties | 5 | 0.151 | 1535 | huangbacha laofu | Local varieties | 4 | 0.273 |
| 757 | pobeihong | Local varieties | 3 | 0.226 | 1536 | huangjinguEMS-1 | Other germplasm | 4 | 0.540 |
| 758 | baisigu | Local varieties | 3 | 0.294 | 1537 | huangjinguEMS-2 | Other germplasm | 4 | 0.239 |
| 759 | dayinggu | Local varieties | 3 | 0.270 | 1538 | huangjinguEMS-3 | Other germplasm | 4 | 0.237 |
| 760 | daguzi | Local varieties | 5 | 0.054 | 1539 | huangjinguEMS-4 | Other germplasm | 4 | 0.128 |
| 761 | xiaojiehuang | Local varieties | 3 | 0.025 | 1540 | huangjinguEMS-5 | Other germplasm | 4 | 0.417 |
| 762 | banhonggu | Local varieties | 3 | -0.207 | 1541 | huangjinguEMS-6 | Other germplasm | 4 | 0.196 |
| 763 | luojingu | Local varieties | 3 | 0.349 | 1542 | huangjinguEMS-7 | Other germplasm | 4 | 0.135 |
| 764 | hongmiaolaodan | Local varieties | 3 | 0.174 | 1543 | huangjingufushe | Other germplasm | 4 | 0.369 |
| 765 | laolaijiao | Local varieties | 3 | 0.027 | 1544 | huangbacha | Local varieties | 4 | 0.354 |
| 766 | liangbadou | Local varieties | 3 | 0.280 | 1545 | huangjingufushe | Local varieties | 4 | 0.334 |
| 767 | longshegu | Local varieties | 3 | 0.113 | 1546 | xinglonggouhuangjingu | Local varieties | 4 | 0.134 |
| 768 | tanshanggu | Local varieties | 3 | -0.007 | 1547 | xianggu | Local varieties | 4 | 0.427 |
| 769 | chaogu | Local varieties | 1 | -0.102 | 1548 | huangjingubufennie | Local varieties | 4 | 0.149 |
| 770 | ruansanshi | Local varieties | 5 | 0.220 | 1549 | aohanhuanggu | Local varieties | 4 | 0.179 |
| 771 | maohonggu | Local varieties | 2 | -0.276 | 1550 | aohanhuanggu | Local varieties | 4 | 0.394 |
| 772 | laolaisonggu | Local varieties | 5 | -0.050 | 1551 | huangjingubufencha | Local varieties | 4 | 0.340 |
| 773 | qianchuanbai | Local varieties | 5 | 0.167 | 1552 | huangjingubuyuhoudai | Other germplasm | 3 | -0.119 |
| 774 | jinmiaogu | Local varieties | 4 | 0.192 | 1553 | dajinmiao03 | Local varieties | 4 | 0.149 |
| 775 | shengtoujin | Local varieties | 5 | -0.332 | 1554 | menglongjingu01 | selected variety | 4 | 0.379 |
| 776 | wanjinbian | Local varieties | 3 | 0.405 | 1555 | chigu6 | selected variety | 5 | -0.042 |
| 777 | dabaigu | Local varieties | 3 | -0.049 | 1556 | chigu10 | selected variety | 5 | 0.210 |
| 778 | xiaobaigu | Local varieties | 3 | -0.234 | 1557 | fenggu12 | selected variety | 5 | 0.152 |
| 779 | xiaohuanggu | Local varieties | 2 | -1.077 | 1558 | 6-14 | germplasm strain | 3 | 0.037 |
